# Self-replicating artificial neural networks give rise to universal evolutionary dynamics

**DOI:** 10.1371/journal.pcbi.1012004

**Published:** 2024-03-28

**Authors:** Boaz Shvartzman, Yoav Ram

**Affiliations:** 1 School of Zoology, Faculty of Life Sciences, Tel Aviv University; Tel Aviv, Israel; 2 School of Computer Science, Reichman University; Herzliya, Israel; 3 Sagol School of Neuroscience, Tel Aviv University; Tel Aviv, Israel; 4 Edmond J. Safra Center for Bioinformatics, Tel Aviv University; Tel Aviv, Israel; University of Zurich, SWITZERLAND

## Abstract

In evolutionary models, mutations are exogenously introduced by the modeler, rather than endogenously introduced by the replicator itself. We present a new deep-learning based computational model, the *self-replicating artificial neural network* (SeRANN). We train it to (i) copy its own genotype, like a biological organism, which introduces endogenous spontaneous mutations; and (ii) simultaneously perform a classification task that determines its fertility. Evolving 1,000 SeRANNs for 6,000 generations, we observed various evolutionary phenomena such as adaptation, clonal interference, epistasis, and evolution of both the mutation rate and the distribution of fitness effects of new mutations. Our results demonstrate that universal evolutionary phenomena can naturally emerge in a self-replicator model when both selection and mutation are implicit and endogenous. We therefore suggest that SeRANN can be applied to explore and test various evolutionary dynamics and hypotheses.

## Author summary

Computational self-replicators are often modeled with implicit definitions of fitness, using various approaches such as biophysical modelling, molecular dynamics, ecological dynamics, and computational logic. However, mutation is usually explicitly defined using standard probability distributions such as the Poisson distribution. Here, we attempted to develop a computational self-replicator with implicit fitness and mutation processes using artificial neural networks, which are popular, complex, and flexible models for approximating solutions to a wide range of computational problems. The result is the *self-replicating artificial neural network* (SeRANN), which learns to copy its own genotype while simultaneously solving a computational problem such as image classification. Approximation errors implicitly introduce mutations, which affect the source code of the networks, changing network hyper-parameters (e.g, number of neurons in a layer), network architecture (connections between layers), and hyper-parameters of the learning algorithm. We evolved a population of SeRANNs and observed various evolutionary phenomena often seen in evolutionary experiments with microbes, demonstrating that this new evolutionary framework provides a promising model for further studies in evolutionary theory.

## Introduction

Evolution by natural selection requires the generation of heritable variation in traits that determine the reproductive success of individuals. In biology, this requirement is satisfied by error-prone replication of genetically determined traits. Mathematical models, e.g., Fisher’s geometric model [1], apply explicit rules and functions both to determine fitness and to generate heritable variation, for example using a pre-determined mutation rate and a specific parametric distribution of fitness effects. Even in studies that use sophisticated artificial-life models such as *Avida* [2] and *aevol* [3] to test evolutionary hypotheses, reproductive success is implicit to the model, whereas replication is still mostly explicit. In the field of *neuroevolution*, artificial neural networks (ANNs) are used as individuals in evolving populations [4]. Reproductive success is implicitly determined by the ANN performance on a task: it emerges from the complex interaction between the task (e.g., classification) and traits of the ANN (e.g., type and number of network layers, number of neurons in each layer). However, replication is still explicit: the ANN genotype (i.e., genetic encoding) is copied by a genetic algorithm, rather than by the ANN itself, and this algorithm adds new mutations to the replicated genotype following rules defined by the modeler/algorithm developer [5].

There are major shortcomings to this approach for modelling evolution with explicit and externally defined replication mechanisms. Models are limited by their assumptions. Hence, models based on (sometimes simplistic) assumptions on how genetic variation is generated in living organisms are limited to pre-defined trajectories and outcomes. This includes models for studying evolution in general, and particularly models for studying the evolution of the replication mechanism (evolution of the mutation rate, evolution of sex, etc.).

We propose that, like reproductive success, replication can also be internalized by ANNs; that is, an ANN can approximately copy its own genotype. Therefore, both replication and reproductive success can be implicit and endogenous, thereby avoiding the standard assumptions taken by evolutionary models and genetic algorithms, such as uniform randomness or separation between alleles responsible for survival and reproduction and alleles responsible for replication [6].

Existing computational evolutionary frameworks have produced exciting results. *Avida* [2] has been used to study the evolution of mutational robustness [7], complex traits [8], the mutation rate [9], sexual reproduction [10], genome architecture [11], drift robustness [12], and more. In *Avida*, organisms are modeled by computer programs: sequences of instructions in an assembly-like language. The instructions are processed by a CPU with three registers, two data stacks, and I/O buffers. Each organism is assigned CPU time according to its fitness, which is determined by its performance on Boolean logic operations (e.g., correctly computing XOR), and therefore the emerging fitness landscape is rugged. Genetic variation is mostly introduced at a rate explicitly determined by the modeler via copy errors and indels that occur during replication and point mutations that occur during the individual lifetime. Implicit mutations can occur during replication due to an incorrect copy algorithm in the parent program, but these mutations are rare, often lethal, limited in their effect (i.e., no copy errors), and can therefore be turned off by the modeler using a configuration option [2,13]. Another computational framework, *aevol*, has been used to study the effects of mutation rate [14,15], pleiotropy [3], and selection intensity [16] on genome architecture. In *aevol*, genomes are modeled by a double-strand binary string, with specific signals for start and stop of coding sequences, which are transcribed and translated to a triangular function (the protein). These triangular functions are defined by their center, width, and height (which are encoded by the coding sequence), and are combined to produce the phenotype. Fitness is then determined by the fit of the phenotype function to a target environmental function, creating a complex fitness landscape. Genetic variation is produced by point mutations, indels, duplications, translocations, and inversions, all randomly drawn from a set of explicit distributions defined by the modeler.

These computational self-replicators are based on particular computational paradigms. Importantly, ANNs are generic and flexible machine learning models that can approximate a very wide range of complex functions and programs. Therefore, an evolutionary framework based on ANNs can allow to compare and contrast multiple computational paradigms by modifying the ANN architecture (CNN, LSTM, Transformer, etc.), the problem domain (vision, language, decision making, etc.), and the training or optimization strategies (flavors of stochastic gradient descent, MCMC, variational inference, etc.), to determine if and how these specific choices affect the evolutionary dynamics.

**Fig 1:**
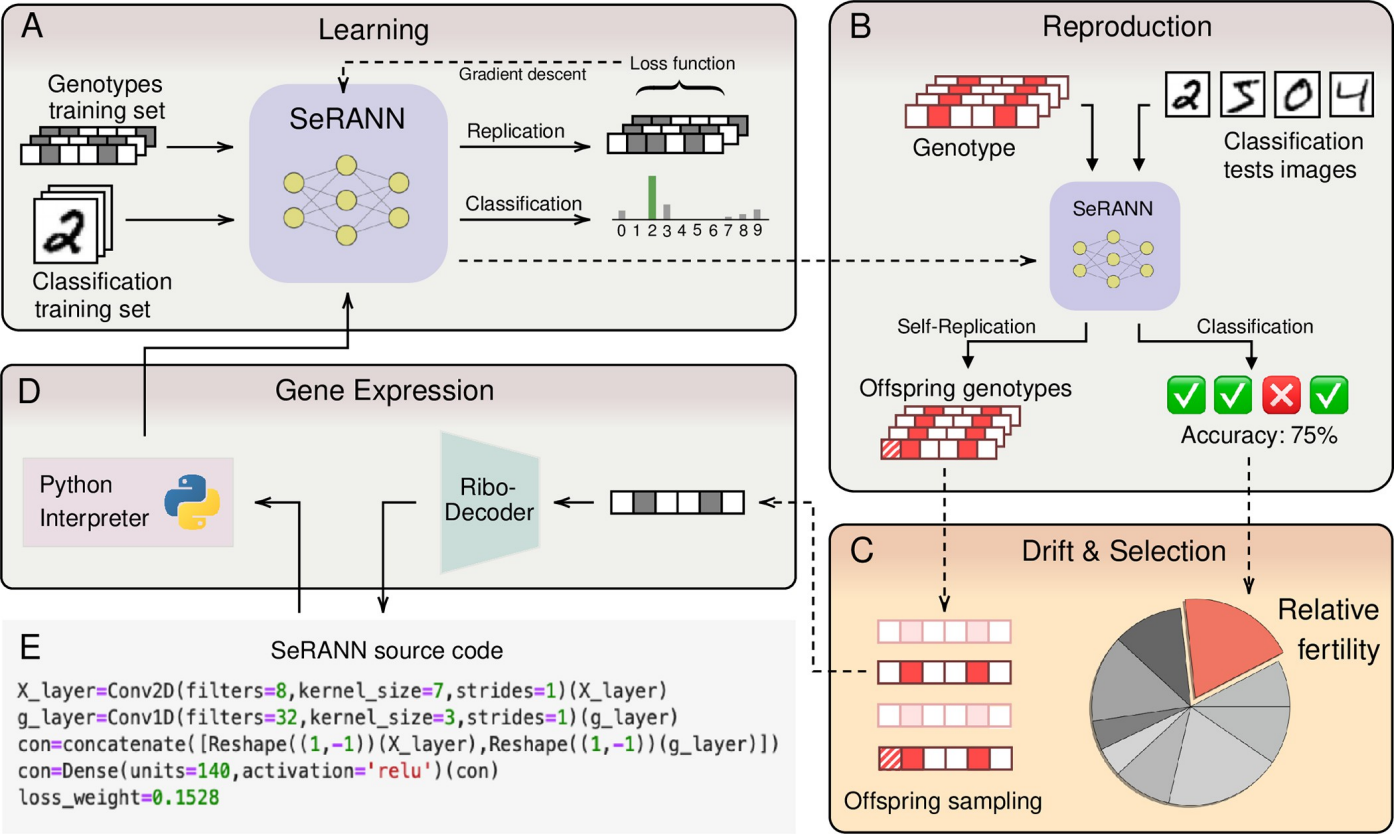
SeRANN evolution framework. **(A)** A juvenile SeRANN individual “learns” to both classify images from the training set and to copy arbitrary genotypes using standard deep-learning techniques. **(B)** An adult SeRANN then classifies images from the test set and copies its own genotype, producing a classification accuracy, which is its fertility, and replicated genotypes, which are its offspring genotypes. **(C)** The individual fertility is compared to the population mean fertility to determine the individual’s expected contribution to the offspring generation–which are then sampled from the offspring genotypes of all individuals. **(D)** Each genotype is decoded to a source code using the *RiboDecoder* (see Methods) and executed by the Python interpreter. Only valid source codes, which don’t cause execution errors (e.g., due to syntax errors), continue to the next generation. **(E)** The source code of the ancestor of the population, see Fig B in S1 Text for the genotype and supporting code.

We present a new kind of computational self-replicator: the *self-replicating artificial neural network*, or SeRANN (Fig 1). Its genotype is a bit-string of 100 bits that encodes its phenotype, which is a *Python* [17] source code of arbitrary length. SeRANNs “learn” (in the sense of *machine learning*) to faithfully copy their own genotypes and simultaneously solve an independent computational task that determines their fertility, a component of reproductive success. Hence, both reproductive success and replication are implicit and endogenous: errors in the replication task implicitly introduce mutations to offspring genotype, which can introduce heritable modifications to offspring phenotype. Notably, only neural-network architecture and hyper-parameters are defined in the source-code phenotype and are thus inherited via the genotype and allowed to evolve, whereas network parameters (i.e., weights) are not inherited, but rather found by “training” or “learning” with standard deep-learning techniques.

The SeRANN genotype-to-phenotype map or “genetic code” is implicitly defined by a separate, custom-built artificial neural network, the *RiboAE*, which we pre-trained to efficiently translate Python source code to bit-strings and back (see *Methods*, Figs A and B in S1 Text). During its training, the RiboAE learned a robust genetic code that results in a smooth fitness landscape: small changes in the genotype (mutations) usually result in minor or even no phenotype modifications, and lethal mutations are unusual (though not rare), similar to *translational robustness*, which in biological organisms relates to the ability of proteins to fold properly despite missense mutations [18].

We evolved a population of SeRANNs for 6,000 generations using a Wright-Fisher framework with non-overlapping generations and a constant population size of 1,000 individuals. At each generation (Fig 1), the number of offspring of each individual is determined according to its performance on an image classification task (*fertility selection*), which represents a cognitive task related to reproductive success. The genotypes of its offspring are copied by a self-replication (i.e., reconstruction) task, such that replication errors modify the offspring genotype, producing heritable variation (*mutation*). The next generation is randomly sampled from the offspring pool (*genetic drift*). Offspring genotypes are then translated (i.e., decoded) to source-code phenotypes and executed by the Python interpreter (*gene expression)*. Only offspring with a valid source code that does not produce execution errors are retained (*survival selection*). Hence, SeRANN *fitness* is the product of *fertility*, which represents success in the classification task, and *survival*, which represents success in the replication task. Surviving offspring are “trained” using gradient descent and backpropagation with *Keras* [19], a deep-learning library, producing the next adult generation (*learning*). Each individual is “trained” on a set of 57,000 classification examples from the well-studied MNIST handwritten digits dataset [20] and 57,000 genotypes from our synthetic dataset (see *Methods*) for five epochs (full passes over the training set). It is then evaluated on a separate test set of 3,000 classification examples and its own genotype to determine its fertility and generate its offspring genotypes. Because a SeRANN has two tasks, classification and replication, the loss function used for “training” a SeRANN is a weighted average of the loss functions of the two tasks. The loss_weight, which determines the relative importance of the two tasks, is a hyper-parameter that is defined in the SeRANN source code (Fig 1E). Thus, it is inherited through the genotype and evolves like a modifier allele [21], allowing the mutation rate itself to evolve.

In the following sections we (i) provide a detailed description of SeRANN, RiboAE, and the evolutionary framework, (ii) present the results of a 6,000-generation evolutionary experiment, and (iii) conclude with a discussion of our framework and results.

## Methods

We introduce a new framework for *in-silico* evolutionary experiments with populations of self- replicating artificial neural networks, or SeRANNs. The genotype of a SeRANN is a bit string, a sequence of zeros and ones. The phenotype of a SeRANN is a Python source code that implements the artificial neural network and can therefore be trained and evaluated like any other machine-learning algorithm (Fig 1).

Importantly, spontaneous replication errors produce genetic mutations, thus providing a source of genetic variation that can manifest to phenotypic variation. Replication errors may in some cases even affect offspring survival due to lethal mutations that produce invalid source code (e.g., Fig L in S1 Text). Fertility is determined by the performance of the SeRANN on an independent task: MNIST image classification. We performed a long-term *in-silico* (simulated) evolutionary experiment with a population of 1,000 SeRANNs, analyzed the emerging dynamics, and compared them to dynamics from lab experiments with populations of living organisms.

### SeRANN model

Formally, a SeRANN is an artificial neural network that approximates a function $\left(\hat{y},g\prime )=F(X,g\right)$, such that

$$F:\;R^{m\times n}\times {\left\{0,1\right\}}^{k}\to {\left[0,1\right]}^{L}\times {\left\{0,1\right\}}^{k}.$$

The inputs are X, a grayscale image with m = 28 rows and n = 28 columns of pixels, and g, a genotype bit-string with k = 100 bits (i.e. zero or one values). The outputs are $\hat{y}$, a probability vector of size L = 10, where each entry ${\hat{y}}_{i}$ in this vector represents the probability that the image X is of the i-th class ($\sum_{i=0}^{L-1}{\hat{y}}_{i}=1$); and g′, a bit-string genotype, also with k bits. See Fig 1 for an illustration.

The objectives of the SeRANN are (i) to accurately classify X to the correct class, by giving the highest probability ${\hat{y}}_{i}$ for the correct class i, and (ii) to faithfully replicate g, such that g′ is as similar as possible to g. A SeRANN is trained using stochastic gradient descent with backpropagation, a standard practice in deep learning [22] with the Adam optimizer [23]. The network’s (trainable) parameters are randomly initialized before training using Glorot initialization [24]. This initialization scheme is commonly used to prevent exploding and/or vanishing gradients and to accelerate training convergence. We define two loss functions. First, ℓ_*X*_ is the categorical cross entropy between the ground-truth vector y and the predicted probability vector $\hat{y}$,

$$l_{X}(y,\hat{y})=-\sum_{i=0}^{L-1}y_{i}\log\left({\hat{y}}_{i}\right).$$

Second, ℓ_*g*_ is the mean squared error between the input genotype *g* and the output genotype *g’*,

$$l_{g}(g,g^{\prime })=\frac{1}{k}\sum_{j=0}^{k-1}{\left(g_{j}-g_{j}^{\prime }\right)}^{2},$$

where *g*_*j*_ is the *j*-th bit of genotype *g*. The loss function ℓ used for training SeRANNs is a weighted average of the above loss functions,

$$l(y,\hat{y},g,g^{\prime })=\alpha \cdot l_{X}(y,\hat{y})+(1-\alpha )\cdot l_{g}(g,g^{\prime })$$ (1)

where the weight 0≤*α*≤1 is determined by a SeRANN source-code variable, loss_weight. Hence, mutation and selection can change the value of this variable, which effectively controls how much of the training effort is dedicated to each of the two tasks.

### Source code representation

We implement SeRANN using the Python programming language [17] and the Keras deep-learning library [19]. This library provides a simple API for defining, training, and evaluating artificial neural networks. In general, each line describes a layer in the network, including the layer type, its hyperparameters and the previous layer it is added upon.

The full SeRANN source code includes the loading of necessary packages (import statements); declaring constant experiment parameters (classification image shape, genotype length); the SeRANN layers definition; and the initialization of the network model object. Of these, only the SeRANN layers definition varies between different SeRANN individuals. Therefore, the SeRANN genotype only encodes this part of the source code, see lines 9–14 in Fig B in S1 Text, which shows a full example source code of a SeRANN implementation.

### Genetic code and ribosomal autoencoder

In biology, the *genetic code* is used to translate (i.e. decode) information encoded in the genetic material (DNA or RNA molecules) to proteins, which, through interactions with the biotic and abiotic environment, give rise to the phenotype. This translation is done by the ribosome. Likewise, we use a genetic code to translate a bit-string genotype *g* to a Python source-code string (Fig B in S1 Text). This source code is then interpreted by the Python interpreter to an instance of a neural network model implemented in Keras, and trained by supervised learning, gradient descent, and backpropagation, to produce the”adult” phenotype.

The viable SeRANN phenotypes space is constrained: SeRANN source code must be exe- cutable by Python interpreter. This means that the genotypes space topology and the genotype- phenotype encoding must be designed with a special care. Otherwise, the vast majority of mutants (i.e. errors in replicated genotypes) would be doomed to immediate “death”, as their translation will suffer from invalid syntax and fail to execute. The fitness landscape induced by such flawed encodings would be sparse, or”perforated”—many potential solutions will be virtually unreachable and the efficiency of the evolutionary process will reduce [25]; see also “holey” adaptive landscapes [26].

To translate genotypes to source codes, we introduce the *ribosomal autoencoder* (*RiboAE*). The RiboAE is an entirely separate neural network from SeRANN. We have trained it to encode Python source code to a genotype bit-string, and faithfully decode it back to source code, using unsupervised learning, which only requires examples of source codes, without examples of genotypes. After training the RiboAE, it was kept constant, and did not change during SeRANN evolution.

### SeRANN source-code generator

To generate a significant number of synthetic SeRANN source-code examples, sufficient for training the RiboAE, we implemented a heuristic SeRANN source code generator. This generator is a stochastic state machine (i.e., Markov chain) that defines the probability of transition between a limited set of manually selected ANN layers (see Table D in S1 Text). For example, given that the current layer is a convolutional layer, the next layer will be a pooling layer with probability 0.7, another convolutional layer with probability 0.2, or a fully-connected layer with probability 0.1.

The hyper-parameters of each network layer were drawn from a discrete normal distribution around reasonable expected values. For example, we sampled the number of neurons in a fully-connected layer from a normal distribution, *N(μ = 64*,*σ = 15)*, clipped the values to lay between 8 and 128, and rounded them to the nearest integer.

To ensure the validity of the generated SeRANN source codes, we enforced a general structure: a fixed-size input layer, a merge layer (to join both classification and replication input branches) and a fixed-size output layer. Thus, we allowed SeRANN source-code examples to diverge by the number of hidden layers, and their types and hyper-parameters, while keeping them compatible with the replication and classification tasks. We used the SeRANN generator to generate 1,000,000 examples of SeRANN source codes.

### RiboAE architecture and training

The RiboAE takes a neural network source code as input and is trained to output the exact same source code (Fig A in S1 Text). The *d*-dimensional input and output of the RiboAE is a SeRANN Python source-code string (Fig B in S1 Text), segmented into a sequence of atomic language tokens. These tokens include variable names and literals (digits, brackets, etc.). The sequence of tokens is padded to exactly *d* tokens using a special padding token. Tokens are mapped to integers according to a predefined vocabulary (see complete vocabulary in Table A in S1 Text).

The RiboAE is composed of two components: the *RiboEncoder* and the *RiboDecoder*. The RiboEncoder consists of an embedding layer, three convolutional layers, and a fully connected layer. It takes as input a *d*-dimensional sequence that represents the source-code string as *d* tokens, and encodes it to a binary *k*-dimensional genotype vector *g*, where *k ≪ d*. This is parallel to the molecular process of *reverse translation* (note that reverse translation and reverse transcription are different processes). The RiboDecoder consists of a single convolutional layer and a fully connected layer. It takes a binary *k*-dimensional genotype vector *g* as input and reconstructs the original *d*-dimensional source-code input, by estimating the categorical probabilities for each of the *d* tokens. This is parallel to the molecular process of *translation*.

In practice, the output of the RiboEncoder is a *k*-dimensional continuous variable, *ϕ*, where each of its values (*ϕ*_1_, *ϕ*_2_,…,*ϕ*_*k*_) is in the interval *[0*,*1]*. During training, *ϕ* is not provided directly to the RiboDecoder, but rather interpreted as parameters of a *k*-dimensional Bernoulli distribution. A random sample from this distribution is used as input to the RiboDecoder.

By training it to overcome minor replication errors, this random sampling procedure allows the RiboDecoder to become robust to mutations. It also reduces the number of lethal “holes” in the genotype space, as reflected by the distribution of fitness effects of new mutations. The fraction of lethal mutations in our main experiment is 10%-20% lower than the one estimated for viruses and yeast. Because the RiboAE is trained using backpropagation and gradient descent [22] with the Adam optimizer [23], the aforementioned random sampling poses two problems: backpropagation through random nodes, and gradient descent with discrete variables, which are not differentiable. We solve the first problem using the reparameterization trick [27]: we transform a sample from a uniform distribution rather than directly sampling from a Bernoulli distribution. Thus, the RiboAE reconstruction error can backpropagate through the RiboDecoder and *ϕ* to the RiboEncoder.

The second problem arises because we would like our genotypes to be consistent with natural genotypes, which are discrete and consist of combinations of four nucleotides, A, G, C, and T. Here, we use binary genetics (0 and 1), although other genetic systems such as quaternary genetics (A, G, C, and T) are possible in future work. We solve the problem of non-differentiability using the *Concrete distribution*, a “relaxed” version of the Bernoulli distribution [28]. For each output of the RiboEncoder, *ϕ*_*i*_, we sample a value from a continuous uniform distribution, *u*_*i*_ *∼ U(0*, *1)*, and use the reparameterization trick, followed by a sigmoid transformation (i.e. logistic function) to produce the genotype value *g*_*i*_,

$$z_{i}=log\phi_{i}+logu_{i}-log(1-u_{i})$$

$$g_{i}=\frac{1}{1+e^{-z_{i}}}.$$

The resulting vector, *g*, consists of continuous values which are mostly very close to 0 and 1. During inference, differentiability is no longer required. Instead of the discrete relaxation, the values of *g*_*i*_ are rounded to 0 or 1,

$$g_{i}=\left\{ \begin{array}{c} 1,\;\phi_{i}>0.5\\ 0,\;\phi_{i}\leq 0.5 \end{array}\right..$$

The RiboDecoder input is the vector *g*. Its output consists of d vectors of length *r*, ${\left\{v_{i}\right\}}_{i=1}^{d}$, where *r* is the number of language tokens in the vocabulary, such that *v*_*i*_ *= (v*_*i*,*1*_,…, *v*_*i*,*r*_*)*. The softmax transformation is applied to each vector *v*_*i*,_ and the result is interpreted as a parameterization of a categorical distribution over the *r* possible language tokens (see Table A in S1 Text for the mapping of tokens to integers). Recalling that *s* is the input token sequence of the RiboAE, and *s*_*i*_ is the integer mapping of the *i*-th token in *s*, the probability of *s*_*i*_ given *v*_*i*_ is

$$P(s_{i}|g)=\frac{\exp\left(v_{i,s_{i}}\right)}{\sum_{l=1}^{r}\exp\left(v_{i,l}\right)}.$$ (2)

The RiboAE is inspired by the variational autoencoder (VAE) of Kingma and Welling [27]. In both cases, the autoencoder is trained to minimize the reconstruction error between the input and output. However, in contrast to the RiboAE, the VAE is simultaneously trained to minimize the Kullback–Leibler divergence between the distribution defined by the encoder output and a prior isotropic distribution (usually a standard normal distribution). Stochastic replication has a biological interpretation: each cell division is equivalent to sampling a genotype from some distribution defined by the original genotype. However, prior constraints on this distribution, as expressed by its divergence from some parametric distribution, have no clear biological interpretation. Furthermore, during preliminary tests we found that introducing the Kullback–Leibler divergence term into the RiboAE loss function added difficulties into the training process.

Thus, the loss function used for the training of the RiboAE, ℓ_*AE*_, is the negative log-likelihood of the input sequence *s*,

$$l_{AE}(s|g)=-\sum_{i=1}^{d}\log\;P(s_{i}|g),$$

where *g* is the result of applying the RiboEncoder on *s* and *P(s*_*i*_ *| g)* is defined in Eq (2).

### Evolutionary model

The evolutionary dynamics in our experiment start with an isogenic ancestral population, made of multiple copies of the same SeRANN. However, our framework also supports diverse ancestral populations, with many different SeRANN. We model an asexual haploid population with constant population size *N* and non-overlapping generations using a Wright-Fisher model. The following steps describe how the next generation of SeRANN individuals is produced from the current generation (Fig 1B).

1. For each individual SeRANN_i_, 1 ≤ *i* ≤ *N*, the source code *s*_*i*_ is interpreted and trained on a classification task ($X\to \hat{y}$) and a replication task (*g → g′*). This step models the processes of development and maturation of the phenotype.
2. Each SeRANN_i_ is given its own genotype *g*_*i*_ and a classification test set of fixed size, *{X*_*j*_*}*_*j*_. For each example *(X*_*j*_,*g*_*i*_*)* in the test set, the mature individual SeRANN_i_ produces a classification prediction and a potential offspring genotype (${\hat{y}}_{i,j},g_{i,j}^{\prime }$), where *j* indexes the test set. Thus, a set of potential offspring genotypes ${\left\{g_{i,j}^{\prime }\right\}}_{j}$ is produced.
  The fertility *F*_*i*_ of SeRANN_i_ is set to its classification accuracy (the fraction of correctly classified examples out of all classified examples). Its relative fertility is set to $f_{i}=\frac{F_{i}}{\sum_{j=1}^{N}F_{j}}$. The relative fertility determines the expected contribution of SeRANN to the next generation in step 4, and thus steps 2–4 model the process of natural selection.
3. The number of offspring of SeRANN_i_ in the next generation *N*_*i*_ is sampled from a multinomial distribution $N_{i}∼Mult(N,\left(f_{1},f_{2},\ldots ,f_{N}\right))$.
4. The genotypes of the next generation are sampled from all the potential offspring genotypes, such that *N*_*i*_ individuals are drawn from the potential offspring ${\left\{g_{i,j}^{\prime }\right\}}_{j}$ of individual *i*. Steps 4–5 model the process of random genetic drift.
5. The RiboDecoder decodes (translates) each offspring genotype *g′* to a new Python source-code string *s′*. Together, steps 2–6 model the processes of replication and reproduction.

The set of decoded source-code strings *s′* is the offspring population, a new population of *N* SeRANN individuals that replaces the parental population. This offspring population is possibly different from the parental population in terms of genotypes and phenotypes due to replication errors (i.e., mutations), selection and genetic drift. To propagate the next generations, we repeat the training, reproduction, replication, and decoding operations in steps 1–6 (Fig 1).

### Experimental setup

We conducted six evolutionary experiments: three with population size *N* = 100, and three with population size *N* = 1,000. Our main experiment, with *N* = 1,000, continued for 6,000 generations. In all experiments, we used genotype length *k* = 100 and SeRANN source code length shorter or equal to 350 language tokens (see Fig A in S1 Text). Genotype length, population size, and number of generations can all be chosen by the modeler, and were chosen in this study to allow for feasible computation time with the resources available to us: we found that longer genotypes required larger neural networks to replicate them, which required more computation time for training; whereas shorter genotypes were too short to faithfully encode even small neural networks. We executed a short preliminary experiment with multiple different ancestors, selected the ancestor that had the most viable descendants after 100 generations, and used it as the ancestor of the population in the main experiment.

Because of the high computational requirements due to the parallel training of thousands of neural networks, we employed GPUs to execute the simulations. For the experiments with *N* = 100 we used a single NVIDIA Titan V GPU. For the experiments with *N* = 1,000, we used a machine with eight NVIDIA Tesla V100 GPUs. An average generation took 9 minutes with *N* = 100 and 17.5 minutes with *N = 1*,*000*. The number of trainable parameters in each SeRANN was limited to 2,000,000 to prevent excessive use of GPU memory. Any SeRANN that exceeded this limit was removed from the population, and its fertility was set to 0. The fraction of such SeRANNs in the population remained very low (about 0.03%), suggesting that the number of parameters did not have a significant effect on networks performance.

For the image classification task that determines the fertility, we used the MNIST handwritten digit image data set [20]. The training set included 57,000 example pairs of handwritten digit image *X* and a synthetic bit-string genotype *g*, and SeRANNs were trained for five epochs (each epoch is one complete pass on the entire training set). The evaluation set included 3,000 example pairs that were not used during training, and fertility was determined according to the SeRANN classification accuracy on this evaluation set.

### Fitness definition

The fitness of a SeRANN individual is determined by two factors. First, the classification accuracy of SeRANN on an evaluation set determines its absolute fertility, *F*. Second, some of the SeRANN offspring will not be successfully interpreted as valid Keras neural networks due to replication errors. The offspring survival rate of a SeRANN, *V*, is thus defined as the probability that its offspring are valid.

Let *F*_*i*_ and *V*_*i*_ be the fertility and offspring survival rate of a SeRANN_i_, respectively. Recall that the relative fertility is

$$f_{i}=\frac{F_{i}}{\sum_{j=1}^{N}F_{j}}.$$

where *j* indexes all individuals in the population. The distribution of the number of newborn offspring of SeRANN_*i*_, *N*_*i*_, is given by a binomial distribution with parameters *N* (population size) and *f*_*i*_,

P(Ni)=(NNi)fiNi(1−fi)N−Ni.

The distribution of the number of *surviving* offspring of SeRANN_*i*_, ${\tilde{N}}_{i}$, conditioned on the number of offspring, *N*_*i*_, is given by a binomial distribution with parameters *N*_*i*_ and *V*_*i*_,

P(N˜i∣Ni)=(NiN˜i)ViN˜i(1−Vi)Ni−N˜i.

Thus, *E*[${\tilde{N}}_{i}$|*N*_*i*_] = *N*_*i*_*V*_*i*_ and *E*[*N*_*i*_] = *N f*_*i*_. From the law of total expectation, the expected number of surviving offspring is

$$E[{\tilde{N}}_{i}]=E[E[{\tilde{N}}_{i}|Ni]]=E[NiVi]=ViE[Ni]=NVi\;fi.$$

Let *p*_*i*_ and *p’*_*i*_ be the frequencies of SeRANN_*i*_ in the current and next generation, respectively. Thus, the relative fitness of SeRANN_*i*_ is the expected change in frequency of SeRANN_*i*_ due to selection,

$$w_{i}=E\left[\frac{p_{i}^{\prime }}{p_{i}}\right]=NV_{i}f_{i}=\frac{V_{i}F_{i}}{\bar{F}},$$

where $\bar{F}$ is the population mean fertility.

However, the relative fitness *w*_*i*_ is unsuitable for comparison of individuals from different generations because of the dependence on the population mean fertility, $\bar{F}$, which changes over the course of evolution. Therefore, we also use the absolute fitness,

$$Wi=V_{i}\cdot Fi.$$ (3)

### Fertility and offspring survival rate estimation

The training process of a neural network generally starts with random parameter initialization. In SeRANN, this initialization can be interpreted as the stochastic aspect of the development and learning processes, which stems from random environmental differences and chance. Two SeRANNs, completely identical in their architecture, may differ in their parameters (network weights) at the end of training. Thus, to accurately assess SeRANN’s properties, such as its classification accuracy or replication fidelity, a single measurement obtained during the exper- iment is insufficient. Moreover, mutations are rare, the number of offspring of each individual is low (1.59 on average), and the genotype length is short (*k* = 100), and therefore estimating the mutation rate from offspring produced during the experiment is likely to underestimate the mutation rate.

Therefore, we performed multiple evaluation cycles of SeRANN properties retrospectively. In each evaluation cycle, the individual SeRANN parameters were initialized from a random distribution, and then the individual was trained and evaluated. The initialization and training steps were identical to those performed during the experiment. After training, the individual classification accuracy was evaluated on 3,000 unseen classification examples (as performed during the experiment). Then, the individual SeRANN was given its own genotype paired with random classification examples and the replicated genotypes were examined to estimate the offspring survival rate and the mutation rate.

The offspring survival rate was estimated by first decoding the replicated genotypes to source codes and testing their validity: each source code was executed by the Python interpreter, together with the required supporting code, and was marked valid only if no errors were raised by the interpreter. The offspring survival rate was then estimated as the fraction of offspring of a genotype that were valid. The mutation rate of a genotype *g* was estimated as the average Hamming distance *H*(*g*,*g’*) between *g* and its offspring *g’* (i.e., the number of sites in which the two genotypes differ), $\mu_{g}=\frac{1}{N_{g}}\sum_{j=1}^{N}H(g,g_{j}^{\prime })$, where *N*_*g*_ is the number offspring of genotype *g*.

### Epistasis estimation

Let *g*_*i*_ be the vector containing the *i*-th site in the genotypes of *h* = 126,158 unique genotypes from our main experiment (*i* = 1,…, *k* and *k* = 100). Let *F*_*g*_ be the fertility (classification accuracy) of genotype *g*. For each pair of sites in genotype *g*, *i*, *j* = 1,…, 100 such that *i*≠*j*, we define the linear regression model for predicting *F*_*g*_ as

$$\hat{F_{g}}=b+a_{i}g_{i}+a_{j}g_{j}+a_{i,j}g_{i}g_{j},$$ (4)

$$F_{g}∼Normal(\hat{F},\sigma_{F}),$$

where *b*, *a*_*i*_, *a*_*j*_, and *a*_*i*,*j*_ are scalar coefficients.

We are especially interested in *a*_*i*,*j*_, which estimates the effect of the interaction between sites *i* and *j* on fertility. Therefore, we also apply a simpler model in which we fix *a*_*i*,*j*_ *= 0*, that is, we assume no interactions between sites *i* and *j*. Using the likelihood-ratio test, we test if the evidence supports a non-zero interaction coefficient. Since we perform 9,900 likelihood-ratio tests (one for each pair of sites in the genotype, which has 100 sites), we adjust the p-values using FDR correction [29].

We fitted these models by minimizing the negative log-likelihood with the least-squares method implemented in SciPy. Fig G in S1 Text shows the estimated interaction coefficients for every pair of sites in the SeRANN genotype.

### Mutational robustness

The mutational robustness of mutations in genotype *g* is given by

$$\lambda (g)=1-\frac{\sum_{g\prime }|W_{g^{\prime }}-W_{g}|\cdot M_{g\to g\prime }}{\sum_{g\prime }M_{g\to g\prime }}$$ (5)

where *g′* iterates over all possible mutants of genotype *g*, *M*_*g→g′*_ is the number of times genotype *g* mutated to genotype *g′* during the experiment, and *W*_*g*_ is the absolute fitness of the genotype *g* (Eq 3), based on 50 classification accuracy measurements and 5,000 offspring survival rate evaluations per genotype. Thus, *λ(g)* is 0 for an optimal genotype (*W*_*g*_ *= 1*) with only lethal mutants (*W*_*g’*_ *= 0*), and 1 for a genotype with only neutral mutations (*W*_*g’ =*_ *W*_*g*_).

## Results

### Overview

We developed SeRANN, a new neural-network-based computational self-replicator with implicit and endogenous replication and reproductive success and explored its evolutionary dynamics. We observed the emergence of universal evolutionary phenomena: changes in both genetic and phenotypic diversity; adaptation by fixation of both synonymous and non-synonymous beneficial mutations; clonal interference, selective sweeps, and genetic hitchhiking between concurrent mutant alleles; epistatic interactions between genetic loci; and a bi-modal distribution of fitness effects. Furthermore, we observed the evolution of the replication mechanism itself: the evolution of a decreased mutation rate and increased mutational robustness. In the following we summarize these phenomena, with additional details provided in the supporting material.

### Adaptation

**Fig 2:**
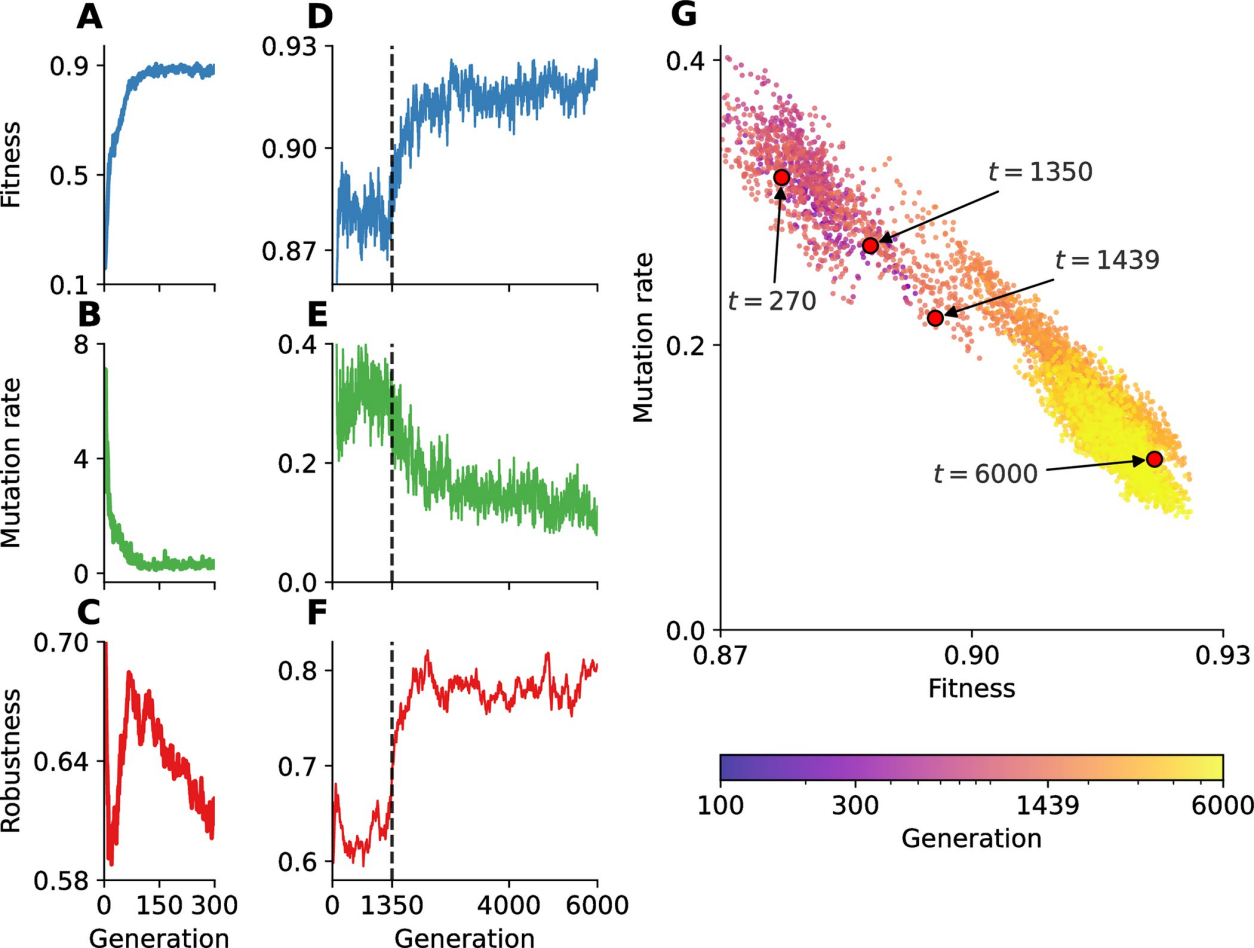
Adaptative evolution. **(A-C)** In the first 270 generations, the population mean fitness increased and the mutation rate decreased, whereas the mutational robustness fluctuated. **(D-F)** Starting around generation 1,350 (dashed line), major adaptations occurred in all three metrics. The number of accumulated mutations increased, but the number of network parameters and the loss_weight value fluctuated rather than trended (Fig H in S1 Text). **(G)** Population mean fitness (x-axis) vs. mutation rate (y-axis) over time (color, brighter is later). Panels D-G show a rolling average over 10 generations. Fitness: survival rate × fertility (Eq 3); Mutation rate: number of mutations per genotype replication; Robustness: one minus average absolute fitness difference between parent and mutant offspring (Eq 5).

Adaptive evolution is clearly demonstrated by the increase in the population mean fitness and the decrease in the population mean mutation rate (Fig 2). Indeed, we expect both to improve over time: fitness due to natural selection favoring fitter genotypes; and the mutation rate due to indirect selection favoring genotypes with lower mutation rates [21,30]. Though it decreased, the mutation rate (measured in mutations per genome per generation) remained orders of magnitude higher than that of viruses and microbes [31] and similar to that of microbial populations with mutator alleles [32], suggesting further improvement could be achieved with more time or longer genotypes.

**Fig 3:**
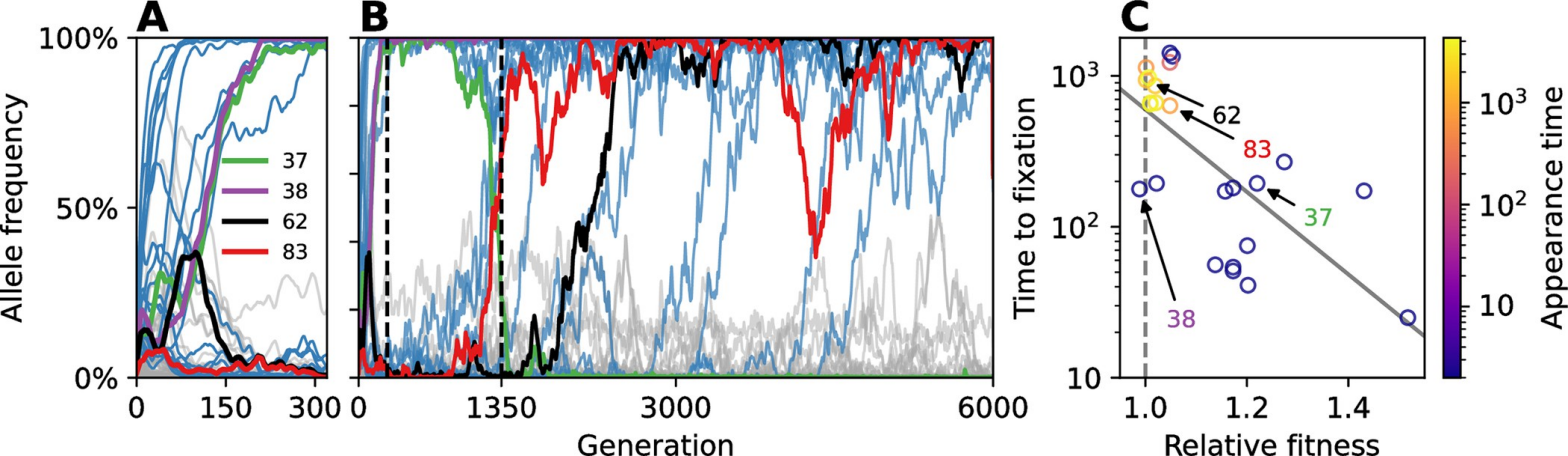
Allele frequency dynamics. **(A)** An initially high mutation rate led to early appearance and fixation of 13 mutant alleles in the first 270 generations. **(B)** Starting from generation 1,350 (dashed line), 11 mutant alleles fixed: four very rapidly, and then roughly one every 585 generations. **(C)** The time to fixation (y-axis) of 24 mutant alleles that successfully fixed (blue lines in A and B) declines with their fitness relative to the population mean at the time they appeared (x-axis; gray line shows linear regression; Pearson correlation *ρ = −0*.*6*, *P < 0*.*002*). Allele 38 (pink) is the only one to reach fixation with relative fitness <1, at 0.988. Gray lines denote mutant alleles that never reached 90% frequency. Frequency curves smoothed for visualization using a rolling average over 10 generations. See Fig J and Table E in S1 Text for details on fixed mutant alleles.

The observed adaptation of the population can be explained by the appearance and fixation of 24 mutant alleles (Fig 3); however, the mutation rate was high enough for the population to remain genetically diverse so that no genotypes fixed during the experiment, and the genetic richness (i.e., number of unique genotypes) remained over 40 (out of 1,000 individuals; Fig E in S1 Text). Of the 24 mutant alleles that fixed, 16 were non-synonymous, that is, they were expressed in architectural and hyper-parameter modifications to the source-code phenotype. The rest were synonymous, with no effect on the source code (Table E in S1 Text), likely the result of genetic hitchhiking [33].

As expected, mutant alleles with larger fitness advantage tended to fix faster (Fig 3C). Moreover, longer fixation times occurred during the initial phase of the experiment (first 270 generations), during which genetic richness was high and 13 mutant alleles fixed, including five synonymous mutants, indicating the effects of genetic hitchhiking [33] and strong clonal interference [34].

**Fig 4:**
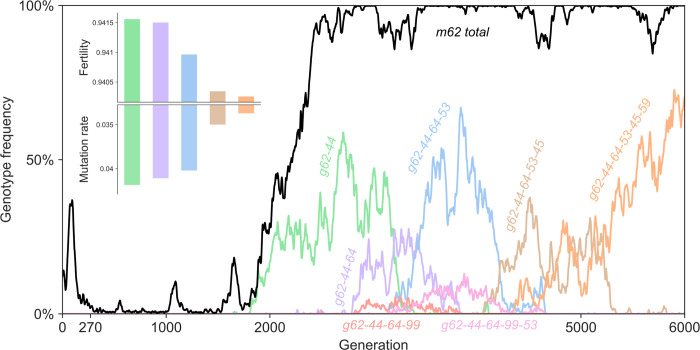
Genotype frequency dynamics: site 62. Due to clonal interference, the total frequency of all genotypes carrying a site-62 mutant allele (black) is much higher than the frequencies of any site-62 genotypes; three genotypes that reached 60% are in green, blue, and orange. The inset shows the fertility and mutation rate of the different genotypes decreasing over time, although the decrease in fertility (0.14%) is minor compared the decrease in mutation rate (19.43%; Table F in S1 Text). These alleles and genotypes appeared more than once during the experiment, in multiple and different parental genotypes (e.g., *m62* occurred 10,702 times during the experiment). Frequency lines smoothed for visualization using a rolling average over 10 generations.

Additional evidence for clonal interference [34] and for soft selective sweeps [35] is given by the dynamics of the mutant allele at site 62 of the genotype (soft sweeps occur when multiple copies of a beneficial mutation, rather than a single copy, appear and fix together). This mutant allele started spreading in the population at generation 1,486, reaching frequency >90% within 940 generations (black line in Fig 3). Three genotypes carrying this mutant allele reached high frequencies (60%-75%) but none fixed in the population (green, blue, and orange in Fig 4). Although these genotypes could be considered an evolutionary lineage, they appeared more than once during the experiment, from multiple and different parental genotypes, and indeed they were not direct offspring of each other. Rather, they were separated by at least two mutation events, and the intermediate mutant genotypes (i.e., the single mutants; purple, pink, peach, and brown in Fig 4) did not reach high frequencies (<15%), as common during adaptation on *rugged fitness landscapes* [36], for example due to *stochastic tunneling*, in which genotypes are part of a successful evolutionary lineage despite never reaching high frequencies [37].

### Diversity

We observed 103,666 unique SeRANN phenotypes (source codes). Surprisingly, the number of unique genotypes was approximately 22% higher than the number of phenotypes, indicating that synonymous mutations, which do not affect the phenotype, are common. The number of unique genotypes in each generation consistently decreased during the experiment (Fig E in S1 Text) likely due to (i) a gradual decrease in mutation rates, which caused less mutations to accumulate; and (ii) selection purging unfavorable genotypes from the population.

### Evolution of the mutation rate

Overall, we observed a significant reduction in the mutation rate. The start of the experiment (first 15 generations) was characterized by a high mutation rate, between 2 and 7 mutations per genotype replication, on average (Fig 2B), due to a high loss_weight value in the ancestor, which controls the balance between the fertility and replication tasks during SeRANN “training”. Therefore, mutant alleles that reduced the loss_weight value in favor of replication contributed to a decreased mutation rate and can thus be considered *anti-mutator alleles*, similar to those found in microbial and viral populations [38]: for example, in the long-term evolutionary experiment with *Escherichia coli*, two *MutY* mutant alleles that appeared on the background of a *MutT* mutator allele reduced the mutation rate by ∼56% and ∼36% [32]. Additional SeRANN anti-mutators appeared later, reducing the mutation rate at the cost of decreased fertility (Fig 4 inset, Table F in S1 Text). In two of these cases, the reduced mutation rate can be explained by a 0.002 reduction in the loss_weight value. The resulting decreases in the mutation rate (1.8–13%) were more dramatic than the decreases in fertility (0.01–0.07%), which may explain the evolutionary success of these of anti-mutators. By the end of the experiment (last 500 generations), the population mean mutation rate was as low as 0.04, a 175-fold reduction compared to its initial value. The variance-to-mean ratio of the mutation rate (across the population) also decreased over time, from ~1.5 at the beginning of the experiment to ~1 at its end. The latter is in agreement with the usual assumption that the number of mutations per genome per generation is Poisson distributed.

The mutation rate was not uniform across the genome. A minority of sites received much more mutations, with a mutation rate above 10^−2^. Interestingly, the sites with the highest mutation rates were located at the two ends of the linear genome (position 0 and 99), which could be explained by the convolutional layer used in SeRANN to process the genome (Fig M in S1 Text).

Furthermore, we tested the effect of providing random images instead of MNIST images on the evaluated mutation rate. We found that the mutation rate with random images was 2.3-fold higher (paired t-test, *P<3×10*^*−6*^, Fig N in S1 Text), and that this increase in mutation rate is correlated with classification accuracy (Pearson correlation, *ρ = 0*.*27*, *P = 0*.*0087*). Therefore, the classification task input (image) affects the performance of the replication task, indicating that the two tasks are dependent, and that unexpected classification inputs can act as “mutagens”.

Surprisingly, we also found a *synonymous* anti-mutator allele in a genotype that reached relatively high frequency (but did not fix; green line in Fig 4). This allele did not affect the source code, but nevertheless had a significant phenotypic effect, reducing the mutation rate by ~16% (*g62-44* in Table F in S1 Text). This synonymous anti-mutator may be compared to the effect of DNA-sequence context on mutation rates [39], such as in CpG hypermutability [40].

**Fig 5:**
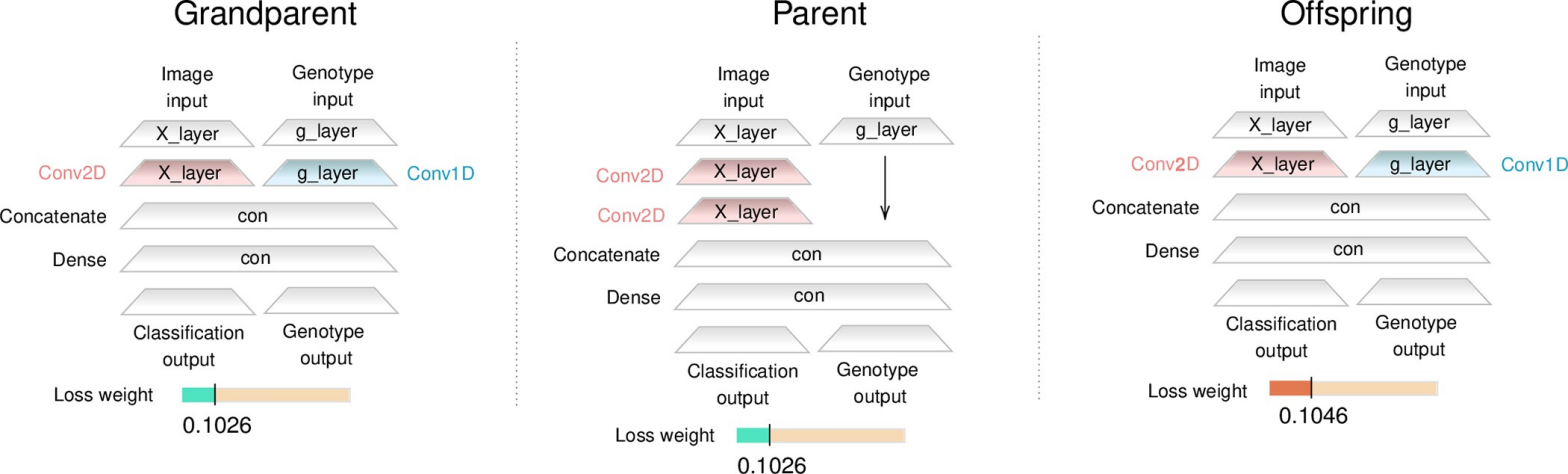
Phenotypic evolution over three generations. The phenotype of three SeRANN individuals from the same lineage over three consecutive generations: “grandparent”, “parent”, “offspring”. The grandparent had 14 mutant alleles (sites 6, 11, 31, 34, 35, 37, 38, 65, 66, 71, 77, 84, 85, 91) compared to the ancestor; these are the 14 mutant alleles that fixed up to generation 1420 (Table E in S1 Text). The parent was born in generation 805 with an additional mutant allele in site 0. This mutation caused a replication layer (g_layer in blue) to switch to a classification layer (X_layer in red). This required the layer names to change, as well as the layer type to change from Conv1D to Conv2D. This modification in the source code had a strong effect on the fertility (up from 0.93 to 0.96), the mutation rate (from <0.02 to 5.5) and the offspring survival rate (down from 0.9988 to 0.063). Despite the low survival rate, the parent managed to produce an offspring in the next generation (805), with 6 additional mutations. Three were reversions back to the ancestor allele at sites 0, 37, and 65. Three were forward mutations to mutant alleles at sites 22, 47, and 83. These three alleles fixed in the population by generation 1439. The effect of the 6 new mutations was to revert the earlier change that occurred in the parent as well as a 0.002 increase in the loss_weight (higher values favor classification). The offspring fertility, at 0.94, decreased compared to the parent, probably due to the layer switch, but increased compared to the grandparent, probably due to the increase in loss_weight (from 0.1026 to 0.1046). The offspring mutation rate, on the other hand, decreased back to a low level (<0.02) and therefore the offspring survival rate increased to 0.9958, much higher than the parent, though not as high as the grandparent. The offspring genotype was the dominant genotype of the three new mutant alleles (sites 22, 47, and 83, see Table E in S1 Text). See Fig D in S1 Text for source codes.

We also identified *mutator alleles*, which increase the mutation rate. For example, a mutant allele that appeared in generation 805 caused a major source-code modification that switched a neural network layer from the replication task to the fertility task (Fig 5). This increased the fertility (from 0.93 to 0.96) but also dramatically increased the mutation rate (from <0.02 to 5.5 mutations per genotype replication). A similar phenomenon was described in *Pseudomonas aeruginosa*, where mutator alleles (*mutS*, *mutY*, and *mutM*) not only increase the mutation rate but also increase fitness by providing resistance to hydrogen peroxide [41], and in *E*. *coli*, where deletion of *mutS* not only produced a mutator phenotype but also extended to delete part of *rpoS*, a gene well known for its adaptive potential under stressful conditions [42].

However, in our case the high mutation rate led to a very low offspring survival rate (0.06, down from 0.99 in the parent). Interestingly, six mutations occurred in one of the mutator offspring, of which three were reverse mutations (i.e., toward the ancestral allele). The combined effect of these six mutations was to revert the major source-code modification described above, as well as to increase the loss_weight value by 0.002 in favor of fertility. The mutation rate thus decreased back to its lower level, although at the cost of an overall 0.02 decrease in fertility (Fig 5).

### Distribution of fitness effects and mutational robustness

**Fig 6:**
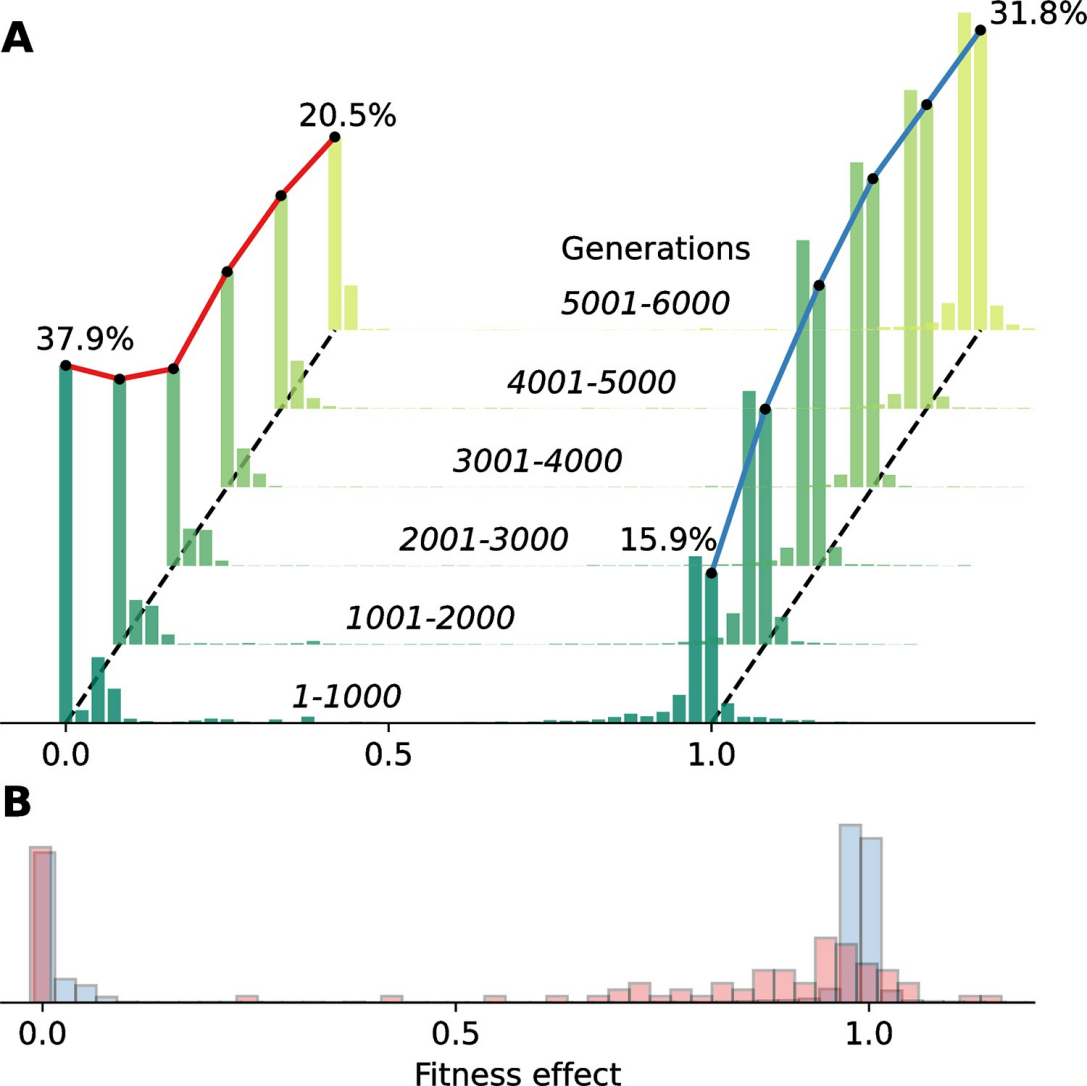
Distribution of fitness effects of new mutations. **(A)** The distribution of fitness effects (DFE) evolved during the *in-silico* evolutionary experiment: the frequency of lethal mutations decreased (red line) and the frequency of neutral and slightly deleterious mutations increased (blue line). **(B)** The DFE in the SeRANN population (blue) is bi-modal with peaks for lethal and near-neutral mutations, similar to a DFE estimated in VSV (red; vesicular stomatitis virus; data from [45]). VSV has more slightly deleterious mutations, whereas SeRANN has more near-lethal mutations, perhaps because the DFE of SeRANN is still evolving. Fitness effects were estimated as the absolute fitness ratio between mutant and parent such that values of 0, <1, 1, and >1 represent lethal, deleterious, neutral, and beneficial mutations. 5,000 parent-mutant pairs were sampled for every window of 1,000 generations for panel A; the average over all generations is shown in panel B.

We accurately measured the fitness effect of every mutation and estimated the distribution of fitness effects of new mutations, or DFE [43]. The estimated distribution is bi-modal and very similar to those estimated in yeast [44] and viruses [45], with high densities of lethal mutations and of neutral or slightly deleterious mutations (Fig 6). We also examined the evolution of the DFE during the experiment. Strikingly, the frequency of lethal mutations decreased over time, whereas the frequency of neutral and slightly deleterious mutations increased (Fig 6). This observed evolution of the DFE is evidence for a phenomenon called “survival of the flattest” [7,46,47]: when the mutation rate is high, as it is in our experiment, the robustness of an organism against lethal mutations is especially important for its survival. Therefore, it is evolutionary advantageous to be in “flat” areas of the fitness landscape, in which mutations have small effects on fitness. Although the fitness in “flat” areas might be sub-optimal, minimizing the effects of mutations on fitness is more advantageous when mutations are common.

Further support for “survival of the flattest” is given by the increase in *mutational robustness*, which is the extent to which a phenotype remains constant in the face of mutations [48,49]. We evaluated the mutational robustness of genotypes that mutated at least once as one minus the expected absolute difference between the parent fitness and the fitness of mutant offspring (see Methods). Overall, the population mean mutational robustness increased over time (Fig 2F) and was positively correlated with the population mean fitness (Fig I in S1 Text). In contrast, Johnson et al. [50] found a negative correlation between fitness and mutational robustness in the yeast *Saccharomyces cerevisiae*. A possible explanation for our observed positive correlation is that lethal mutations became less likely as the experiment proceeded (Fig 6), which increased mutational robustness, but also increased offspring survival rates and therefore the population mean fitness.

### Epistasis

Epistatic interactions between genetic loci were common and had a notable effect on the evolutionary dynamics. This is exemplified by the case of the mutant allele at site 37 of the genotype, *m37*, which reached fixation in generation 195. Its effect on its dominant genotype (the genotype that carried it and had the largest number of descendants) was a reduction in the loss_weight value in favor of replication. Thus, it can be considered an anti-mutator allele. However, *m37* was effectively driven to extinction by generation 1,423 by genotypes that contained the wildtype allele at site 37, *w37*, and a mutant allele at site 83, *m83*, becoming the only allele to fix and then become extinct (specifically, the combination *m37/m83* appeared in generation 804, and in the next generation back-mutations generated the *w37*/*m83* combination).

We examined all genotypes that carried mutant alleles at sites 37 and/or 83 (Table B in S1 Text). We found that when both mutant alleles are present in the same genotype, the average number of neurons (i.e., units) in the next-to-last layer of the SeRANN decreases by 14–17% compared to the average number of neurons when only one mutant allele is present, respectively. As a result, the average mutation rate increases by 2.5 to 5.5-fold. This has a dramatic effect on offspring survival rate, which drops from 93–98% with one mutant allele to 47% with both mutant alleles. Thus, fitness with both alleles is lower than with either allele, leading to *sign epistasis* between these alleles [51]. Because the effect of *m83* on the mutation rate is stronger than that of *m37* and it only requires a minor sacrifice in fertility (Table B in S1 Text), natural selection favors *m83*, leading to its fixation and to the extinction of *m37*.

We also performed a global epistasis analysis of all site pairs in the genotype, focusing on fertility, for which we have greater statistical power. We estimated the epistasis coefficient of all pairs of genotype sites, resulting in a distribution similar to those observed in vesicular stomatitis virus (Fig G in S1 Text) and in *E*. *coli* [52], with roughly half of the coefficients positive and half negative.

### Phenotypic variation

The degree to which the same genotype can develop to different phenotypes under the same environmental conditions is called phenotypic variation. It has been extensively studied in microbial systems, where it can arise from stochastic fluctuations in molecular or cellular processes and attenuated by, e.g., feedback regulation [53]. In SeRANNs, network parameters are randomly initialized before training and therefore multiple SeRANNs with the same architecture trained on the exact same sequence of examples may have different parameters and therefore may differ in their fertility and replication performance.

We measured the variation in SeRANN fertility and offspring survival using repeated cycles of initialization-training-evaluation. We found a wide range of fertility variation (between 0.001 and 0.02; 99^th^ percentile <0.012) and an even wider range of offspring survival rate variation (between 0 and 0.4; median 0.09). Fertility variation positively correlated with average absolute fitness (Pearson correlation, *ρ* = 0.52; permutation test, *P*<10^−4^; Fig F in S1 Text). In contrast, the relationship between variation in offspring survival rate and absolute fitness is more complex. Fitness cannot be close to the neither 0 nor 1 when offspring survival rate variation is high, because fitness is estimated by the average offspring survival rate times the average fertility across all evaluation cycles (Eq 3). Thus, despite a lack of correlation between the standard deviation of offspring survival and absolute fitness (*ρ* = 0.002, *P*>0.73), the fittest SeRANN individuals have low variation in offspring survival (Fig F in S1 Text).

## Discussion

Computational and mathematical models of evolution have been developed and analyzed for roughly a century [54], with crucial contributions to our current understanding of evolutionary biology. However, self-replication, and thus the generation of genetic variation, is usually explicitly defined by the modeler, following her assumptions on the replication process in living organisms. Even when some elements of self-replication are heritable, and can therefore evolve, they are limited to a specific number of pre-determined options, and mutations are usually introduced to the genotype with some probability by a rule-based algorithm [e.g., 55–57]. In parallel, many attempts have been made to design logical self-replicators since the seminal study of von Neumann et al. [58], for example using computer programs [59–61] and artificial neural networks [4]. Artificial neural networks (ANNs) are a promising model for self-replication due to their ability to implicitly solve complex computational problems using efficient learning algorithms running on high-performance graphical processing units (GPUs). Moreover, ANNs have led to the recent explosion in artificial intelligence and are the focus of a vast number of research projects and applications. Thus, an evolutionary framework based on ANNs can benefit from this huge community that includes practitioners across diverse fields, and from the vast number of approaches to construct and train ANNs for various problem domains. However, in previous neural network-based replicators, reproductive success was determined by the network performance on some task, whereas the generation of variation was exogenous to the network and performed by an external genetic algorithm [62,63]. For example, Le Nagard et al. [64] evolved artificial neural networks with a single input and output and up to 20 neurons to study the relationship between task and phenotype complexity. In this model, fitness was implicitly determined by a regression problem (approximating a Legendre polynomial) and mutations were explicitly determined by randomly changing the network weights according to a specified Gaussian distribution. In comparison, our framework uses neural networks with multiple inputs and outputs that allow more complex computational tasks; a large and evolving number of neurons (>1,000); and importantly, self-replication as a distinct computational task, resulting in implicit mutations that affect network architecture rather than network weights, which are learned by backpropagation and gradient descent.

Here we have introduced an endogenous and implicit self-replication process to neural networks in the form of the *self-replicating artificial neural network* (SeRANN). Each network “learns” how to approximately copy its own genotype, and therefore makes “mistakes”, or approximation errors, which implicitly introduce spontaneous mutations to its offspring genotypes. Selection then acts at the population level on the genetic variation produced by these mutations, leading to evolution of network architecture and hyper-parameters. Therefore, as in living organisms, mutagenesis is an emergent process, rather than an external and independent process, that is determined by a complex process and interactions between different sites in the genotype [6]. Moreover, the “learning” algorithm is defined by the SeRANN source code, which can evolve, because it is encoded by the genotype. Thus, indirect or second-order selection [30] leads to a reduced mutation rate, increased mutational robustness, and re-modeling of the distribution of fitness effects (Figs 2 and 6). Note, however, that biological organisms do not “learn” to replicate their genotypes: this element of our framework is not intended to faithfully model biological replication but rather to allow artificial neural networks to implicitly generate genetic variation.

Due to their implicit definitions of both reproductive success and self-replication, SeRANNs can be considered more open-ended [65] than the mathematical and computational models usually used in evolutionary biology and neuroevolution. Moreover, our model does not assume common assumptions that may limit our view of evolution [6], e.g., that mutations are Poisson distributed in time and uniformly distributed throughout the genome. Thus, the genotype-phenotype map is stochastic, complex, and implicit, leading to unpredictable evolutionary trajectories, as is the effect of the phenotype on both the fitness and the mutation rate (e.g., Fig 5). Nevertheless, SeRANN evolutionary dynamics exhibit many of the hallmarks of evolution studied in the experimental and theoretical literature: adaptative evolution is driven by the appearance of new beneficial mutations but hindered by clonal interference, epistasis, pleiotropy, hitchhiking, and drift, which together with soft sweeps, stochastic tunneling, phenotypic variation, and synonymous and reverse mutations give rise to complex evolutionary dynamics at the population (Fig 2), allele (Fig 3), genotype (Fig 4), and phenotype (Fig 5) levels. Our results highlight the universality of these evolutionary phenomena by demonstrating their spontaneous emergence in a population of computational self-replicators with implicit reproductive success and replication.

Our source code is available under an open-access license, and it is straightforward to modify components of SeRANN and the evolutionary framework, for example, by changing the fertility task to another computational task that has a bounded differentiable loss function. In future work, SeRANN can be applied to examine models and methods that have been proposed for studying the genotype-phenotype-fitness mapping, a crucial factor in adaptive evolution [66,67] or to study the evolutionary role of phenotypic variation and developmental noise (Fig F in S1 Text). Another future direction is the evolutionary trade-off between fertility and replication fidelity, which is inherent in SeRANN due to the limited resources (i.e., network parameters) shared by the two tasks of classification and replication. Furthermore, our framework can be used to study potential evolutionary solutions to this trade-off, such as the evolution of a reproductive division of labor: two or more individuals (e.g., offspring of the same parent) aggregate to form a group, and then differentiate to *germ* and *soma*—networks responsible for the replication and classification of the whole group, respectively [68,69]. This could be achieved by adding a hyper-parameter (like the loss_weight variable) that encodes the tendency to aggregate and differentiate. A different future direction is the evolution of social vs. individual learning [70]. In this scenario, an individual-learning SeRANN uses reinforcement learning to update its parameters instead of supervised learning, whereas a social-learning SeRANN uses supervised learning where supervision is given by other individuals (parents, non-parental adults of the previous generation, or even peers) rather than the ground truth. The tendency to be an individual or a social learner can be encoded by a new hyper-parameter and can therefore evolve. These are examples for how SeRANN could provide an intriguing new framework to study questions in evolutionary theory.

## Supporting information

S1 Text Supplementary Figures and Tables. (PDF)
