# Supplementary material for: Self-replicating artificial neural networks give rise to universal evolutionary dynamics
Source: PLoS Comput Biol. 2024 Mar 28;20(3):e1012004. doi: 10.1371/journal.pcbi.1012004 (PMC11003675; doi:10.1371/journal.pcbi.1012004)
Supplement: S1 Text — (PDF) [file pcbi.1012004.s001.pdf]

Self-Replicating Artificial Neural Networks Give Rise to Universal Evolutionary Dynamics

Boaz Shvartzman and Yoav Ram

S1 Text: Supplementary Figures and Tables

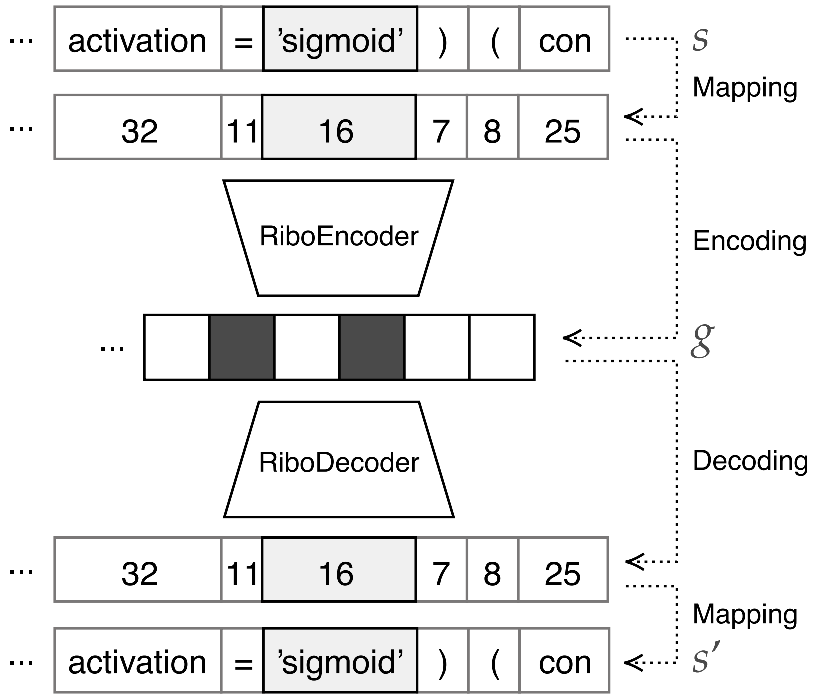

Fig A. Ribosomal autoencoder (*RiboAE*). The *RiboAE* consists of the *RiboEncoder* and *RiboDecoder*. It has been trained to map a sequence of source-code tokens to a bit-string genotype and then map it back to a sequence of source-code tokens. The mapping from source-code tokens to integers and vice-versa is arbitrary and fixed, see table A in S1 Text.

```
Genotype:
0000000111111111000111001001100100001000010100011110010001111010100011010010000111100110001101010111
Source code:
```

```python
1  # Supporting code
2  from keras import Input, Model
3  from keras.layers import Reshape, concatenate, Dense, Conv2D, Conv1D
4  m, n, l, k = 28, 28, 10, 100
5  X_input = X_layer = Input(shape=(m, n, 1))
6  g_input = g_layer = Input(shape=(k, 1))
7  
8  # SeRANN core
9  X_layer=Conv2D(filters=8,kernel_size=7,strides=1)(X_layer)
10 g_layer=Conv1D(filters=32,kernel_size=3,strides=1)(g_layer)
11 con=concatenate([Reshape((1,-1))(X_layer),Reshape((1,-1))(g_layer)])
12 con=Dense(units=140,activation='relu')(con)
13 loss_weight=0.1528
14 
15 # Supporting code
16 y_hat = Dense(l, activation='softmax')(con)
17 g_offspring = Dense(k, activation='sigmoid')(con)
18 model = Model(inputs=[X_input, g_input], outputs=(y_hat, g_offspring))
```

Fig B. Ancestor genotype and phenotype. The bit-string genotype and source-code phenotype of the ancestor of our *in-silico* evolutionary experiment. The neural network implementation is in lines 9-13; this is the part encoded in the genotype. The rest is supporting code, which is not encoded in the genotype. It imports the required objects (lines 2-3), and implements the inputs (lines 4-6), outputs (lines 16-17), and the model (line 18). Source code is written in Python with the Keras deep-learning library.

```
1 # Parent
2 X_layer=Conv2D(filters=8,kernel_size=7,strides=1)(X_layer)
3 g_layer=Conv1D(filters=32,kernel_size=3,strides=1)(g_layer)
4 con=concatenate([Reshape((1,-1))(X_layer),Reshape((1,-1))(g_layer)])
5 con=Dense(units=140,activation='relu')(con)
6 loss_weight=0.1528
```

```
1 # Offspring
2 X_layer=Conv2D(filters=8,kernel_size=7,strides=1)(X_layer)
3 g_layer=Conv1D(filters=32,kernel_size=3,strides=1)(g_layer)
4 con=concatenate([Reshape((1,-1))(X_layer),Reshape((1,-1))(g_layer)])
5 con=Dense(units=180,activation='relu')(con)
6 loss_weight=0.1528
```

Fig C. Example: single mutation. A single mutation at site 71 of the ancestor genotype (as in fig. S2) increased the number of units (i.e., neurons) in the last `Dense` layer from 140 (parent line 5) to 180 (offspring line 5), thereby increasing the number of parameters by *4,400=(180-140)×(10+100)*, as this layer connects to the output layers (lines 16-17 in fig B in S1 Text). This mutation first appeared in generation 1 and fixed in generation 52.

```
# Grandparent
X_layer=Conv2D(filters=32,kernel_size=5,strides=2)(X_layer)
g_layer=Conv1D(filters=8,kernel_size=5,strides=1)(g_layer)
con=concatenate([Reshape((1,-1))(X_layer),Reshape((1,-1))(g_layer)])
con=Dense(units=189,activation='relu')(con)
loss_weight=0.1026
```

```
# Parent
X_layer=Conv2D(filters=32,kernel_size=5,strides=2)(X_layer)
X_layer=Conv2D(filters=8,kernel_size=5,strides=1)(X_layer)
con=concatenate([Reshape((1,-1))(X_layer),Reshape((1,-1))(g_layer)])
con=Dense(units=189,activation='relu')(con)
loss_weight=0.1026
```

```
# Offspring
X_layer=Conv2D(filters=32,kernel_size=5,strides=2)(X_layer)
g_layer=Conv1D(filters=8,kernel_size=5,strides=1)(g_layer)
con=concatenate([Reshape((1,-1))(X_layer),Reshape((1,-1))(g_layer)])
con=Dense(units=189,activation='relu')(con)
loss_weight=0.1046
```

Fig D. Example: multiple mutations over multiple generations. Source code for three SeRANN individuals from the same lineage, here called "grandparent", "parent", "offspring", which constitute a lineage over three generations. The grandparent had 14 mutant alleles (sites 6, 11, 31, 34, 35, 37, 38, 65, 66, 71, 77, 84, 85, 91) compared to the ancestor; these are the 14 mutant alleles that fixed up to generation 1420 (table E in S1 Text). The parent was born in generation 805 with an additional mutant allele in site 0. This mutation caused a replication layer, `g_layer`, to switch to a classification layer, `X_layer`. This required the input and output names to change, as well as the layer type to change from `Conv1D` to `Conv2D`. This modification in the source code had a strong effect on the fertility (up from 0.93 to 0.96), the mutation rate (from <0.02 to 5.5) and the offspring survival rate (down from 0.9988 to 0.063). Despite the low survival rate the parent managed to produce an offspring in the next generation (805), with 6 additional mutations. Three were reversions back to the ancestor allele at sites 0, 37, and 65. Three were forward mutations to mutant alleles at sites 22, 47, and 83. These three alleles fixed in the population by generation 1439. The effect of the 6 new mutations was to revert the earlier change that occurred in the parent, i.e., back from `X_layer` to `g_layer`, as well as a 0.002 increase in the `loss_weight` value. The offspring fertility, at 0.94, decreased compared to the parent, probably due to the layer switch, but increased compared to the grandparent, probably due to the increase in `loss_weight`. The offspring mutation rate, on the other hand, decreased back to a low level (<0.02) and therefore the offspring survival rate increased to 0.9958, much higher than the parent, though not as high as the grandparent. The offspring genotype was the dominant genotype of the three new mutant alleles (sites 22, 47, and 83).

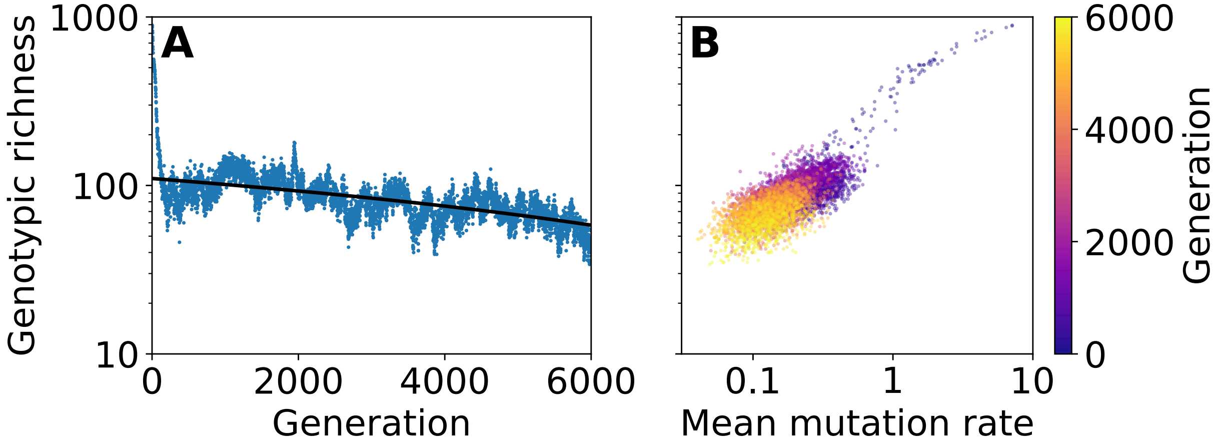

Fig E. Genotypic richness and mutation rate. The genotypic richness (number of unique genotypes) increased to >800 in the first ten generations due to a high mutation rate at ~5 mutations per genotype replication. Both richness and mutation rate then quickly decreased due to selection against deleterious mutations. By generation 100, genotypic richness was at 155 and the mutation rate was at 0.6 mutations per genotype replication. Both continued to decrease: one unique genotype was lost, on average, every 115 generations (linear regression presented by black line in A), whereas the mutation rate decreased by roughly 1% every generation, dropping as low as 0.04 mutations per genotype replication. Pearson correlation between genotypic richness and mutation rate, $\rho = 0.88, P < 10^{-8}$. Mean mutation rate for a given generation is the average number of mutations per genotype replication across all genotypes in that generation.

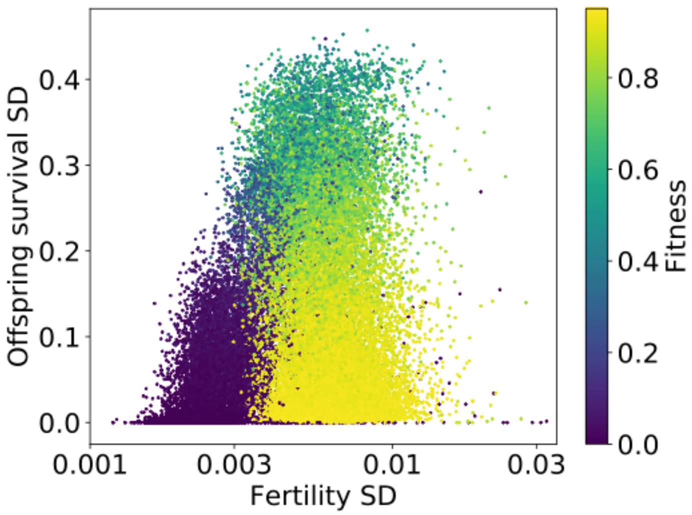

Fig F. Phenotypic variation in fertility and offspring survival. Standard deviation in fertility (x-axis) and offspring survival rate (y-axis) colored by absolute fitness (eq. 3). Fitness is highest (yellow) when fertility variation is high and survival variation is low. Each marker corresponds to one genotype from the evolutionary experiment. Estimated using 50 cycles per genotype, each cycle consisting of parameter initialization, training, and evaluation.

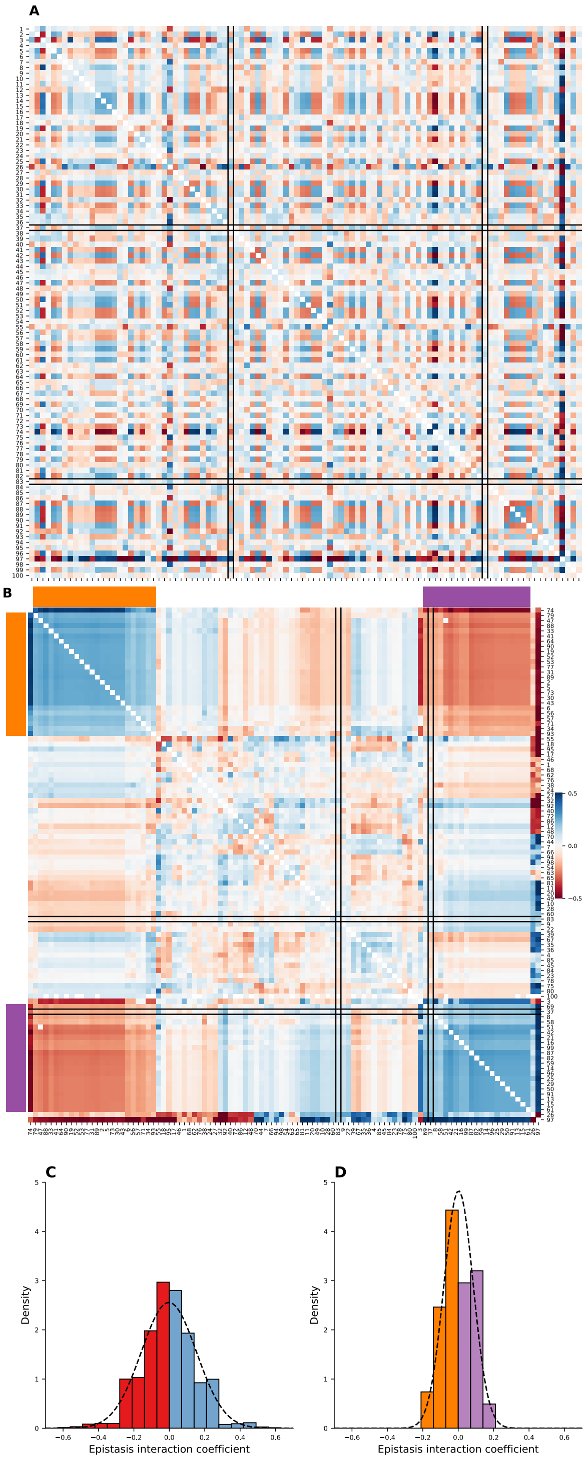

Fig G. The effect of two-site epistasis on fertility. (A-B) The interaction coefficient (eq. 3) for absolute fertility (classification accuracy) for every pair of sites in the SeRANN genotype. Statistically insignificant measurements (*P > 0.05* with FDR correction*)* are set to zero. Sites 83 and 37 are marked with black lines. (A) Rows and columns ordered by genotype site. There are notable interaction "blocks", for example in sites 13-16 and 88-90, which may indicate spatial relationships between adjacent sites in the genotype, likely caused by convolution layers in the *RiboAE* that determines the implicit "genetic code". (B) Rows and columns ordered by hierarchical clustering. There are two large clusters, marked purple and orange, each of about a quarter of the genotype. These clusters show positive within-cluster interactions and negative between-cluster interactions. *m83* and *m37* have negative epistasis according to this global analysis, although not very strong, and they belong to different clusters. **(C-D)** Histograms of the epistasis coefficients from SeRANN (C) and from VSV (D; vesicular stomatitis virus; data from (*14*)). Dashed lines for best-fit normal distributions, C: $N(\mu = -0.00244, \sigma = 0.156)$; D: $N(\mu = -0.00212, \sigma = 0.1456)$. Fraction of negative epistasis, C: 0.512, D: 0.535. Fraction of positive epistasis, C: 0.488, D: 0.465. The difference between the histograms could be attributed to the much larger sample size for SeRANN (*n=9869* significant coefficients) compared to VSV (*n=58*), which allows capturing more extreme values.

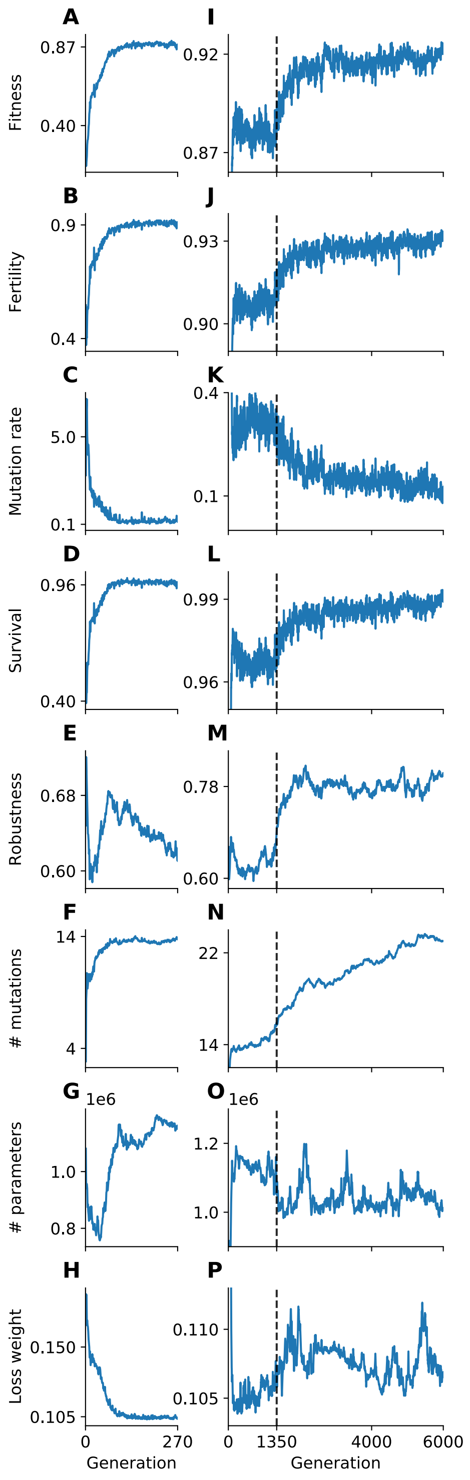

Fig H. Population-level adaptation. (A-H) In the first 100 generations, the population mean fitness, fertility, mutation rate, survival rate, and mutational robustness of new mutations all considerably improved due to the fixation of 13 mutant alleles (Fig 3). **(I-P)** Around generation 1,300 (dashed line), another major improvement occurred in the population metrics. During these adaptation events, the number of accumulated mutations increased, but the number of network parameters and the `loss_weight` value fluctuated, rather than trended. Fitness: survival rate × absolute fertility, eq. 3; Absolute fertility: classification accuracy. Mutation rate: number of mutations per genotype replication; Offspring survival rate: fraction of valid offspring; Robustness: mean absolute fitness difference between parent and mutant offspring, eq. 5; # mutations: number of mutant alleles accumulated in the genotype; # parameters: number of trainable parameters in the artificial neural network; Loss weight: the value of the `loss_weight` variable, eq. 1.

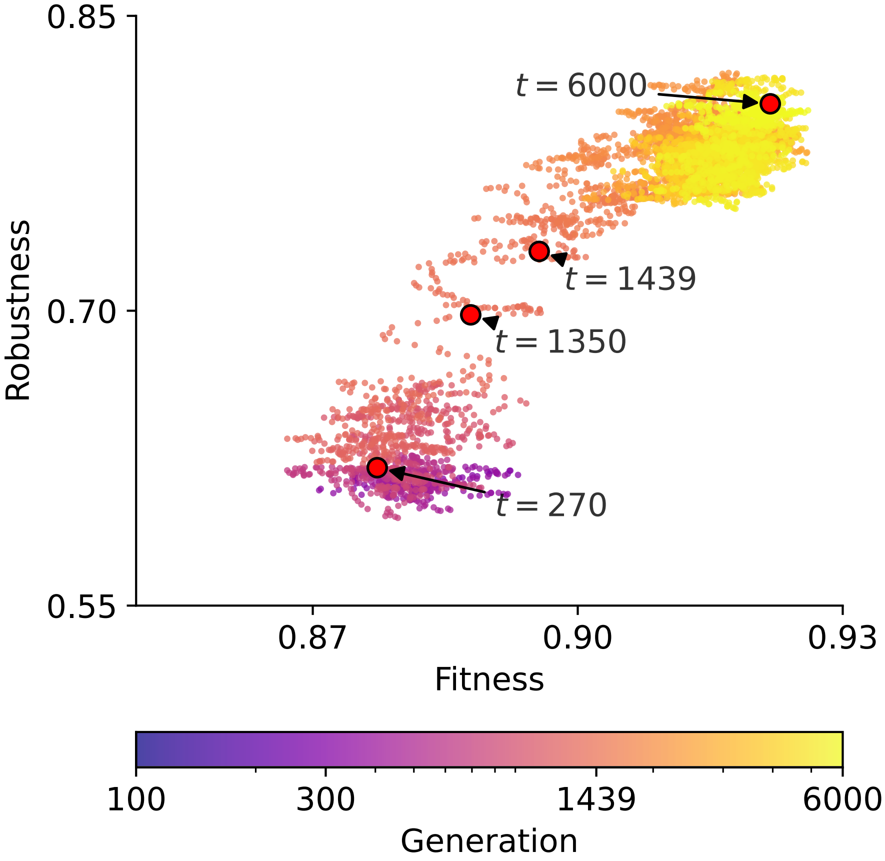

Fig I. Fitness and mutational robustness over time. The population mean fitness (x-axis; eq. 3) and population mean mutational robustness (y-axis; eq. 5) from generation 100 (dark color) to the end of the experiment (light color). A shift towards higher fitness and robustness occurred around generation 1439, during the fixation of three mutant alleles in sites 22, 47, and 83 (fig D in S1 Text; table E in S1 Text). Robustness and fitness are positively correlated: Pearson correlation, $\rho=0.5$, $P\approx 0$.

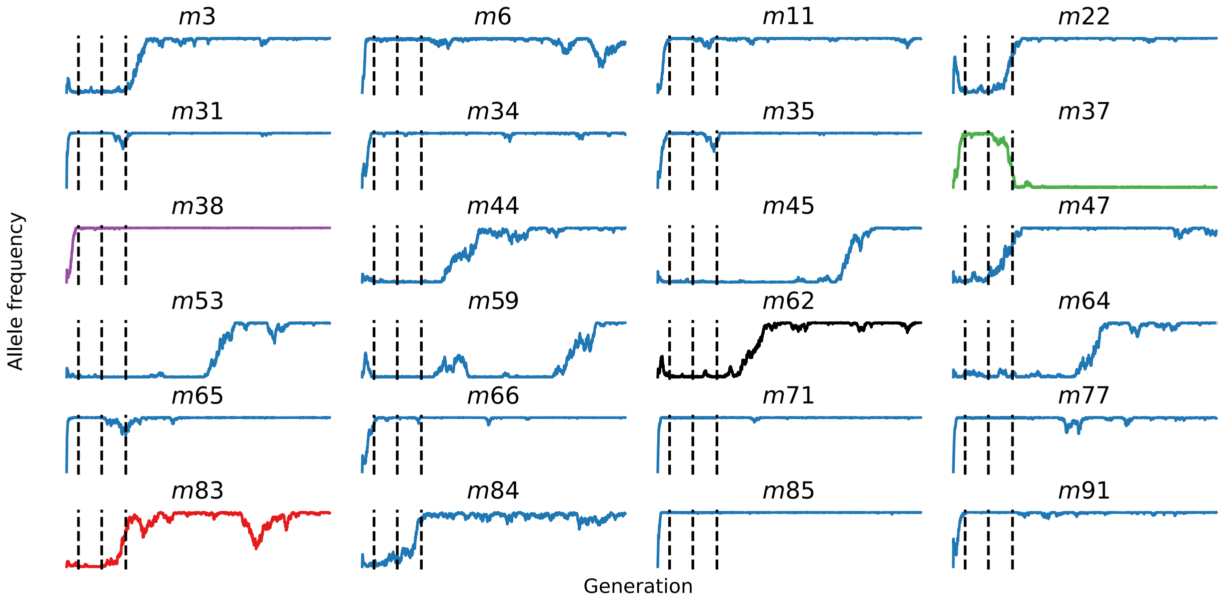

Fig J. Frequency dynamics for 24 alleles that went to fixation. See table E in S1 Text for additional details on these mutant alleles. The x-axis goes from 0 to 6,000 generations; the y-axis goes from frequency 0% to 100%. Line colors correspond to the colors in Fig 3. Vertical dashed lines show generations 270, 800, and 1,350.

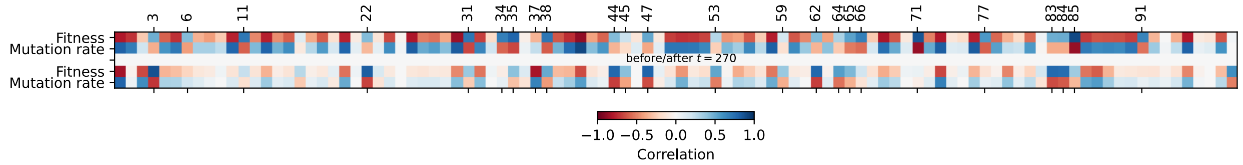

Fig K. Correlation of mutant allele frequencies with fitness and mutation rate. The top two and bottom two rows show the Pearson correlation of the frequencies of mutant alleles before and after generation 270, respectively. Among mutant alleles that went to fixation (table E in S1 Text), the only one showing both negative correlation with fitness and positive correlation with the mutation rate, both before and after generation 270, is at site 37; it is also the only one to become extinct (Fig 3). P-values were corrected using FDR, and insignificant correlations ($P>0.05$) were set as zero; this occurred for just 2.8% of correlations and mostly after generation 270.

```
1 # Parent
2 X_layer=Conv2D(filters=32,kernel_size=5,strides=2)(X_layer)
3 g_layer=Conv1D(filters=8,kernel_size=5,strides=1)(g_layer)
4 con=concatenate([Reshape((1,-1))(X_layer),Reshape((1,-1))(g_layer)])
5 con=Dense(units=117,activation='relu')(con)
6 loss_weight=0.1168
```

```
1 # Offspring
2 X_layer=Conv2D(filters=32,kernel_size=5,strides=2)(X_layer)
3 g_layer=Conv1D(filters=8,kernel_size=5,strides=1)(g_layer)
4 con=concatenate([Reshape((1,-1))(X_layer),Reshape((1,-()))(g_layer)])
5 con=Dense(units=117,activation='relu=)(con)
6 loss_weight=0.1668
```

Fig L. Example: lethal mutation. A single mutation at site 81 of the parent genotype led to an erroneous replacement of the literal “1” by empty parentheses “()” in the offspring source code (line 4, highlighted in red). When the offspring source code is executed by the Python interpreter, an error occurs: “*SyntaxError: invalid syntax*”. Thus, the offspring does not survive and is discarded from the offspring generation.

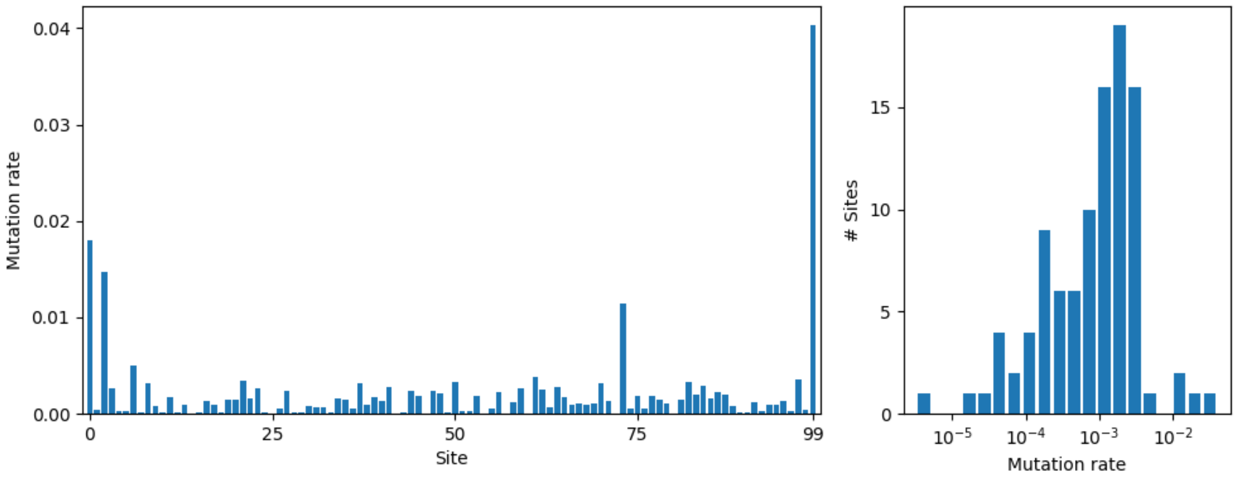

Fig M. Mutation rate across the genome. (Left) The site-specific mutation rate of every position in the genome during the entire evolutionary experiment. **(Right)** Histogram of the site-specific mutation rates.

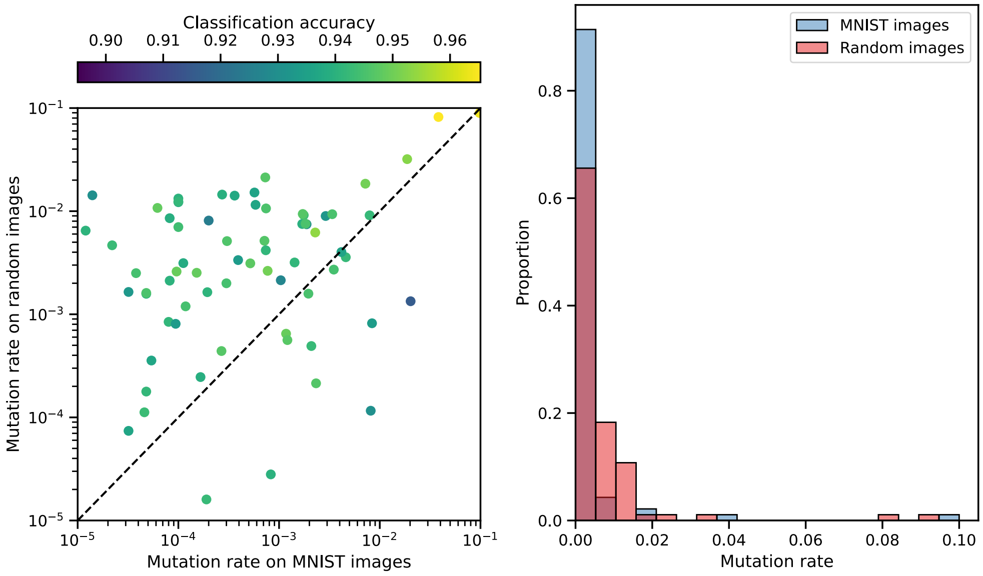

Fig N. Mutation rate with MNIST and random images. The mutation rate of SeRANNs is 2.3-fold higher, on average, when the input is a random image instead of an MNIST image (paired t-test, $P<3\times10^{-6}$). 93 networks were trained 50 times on a random set of genotypes and MNIST images, then averaged over 100 evaluations to determine their classification accuracy and mutation rate. Random images were generated from a uniform distribution. **(Left)** Mutation rate with random images (y-axis) is higher on average than with MNIST images (x-axis). Classification accuracy (color) explains some of the variation in the differences between mutation rates (Pearson correlation, $\rho=0.27$, $P=0.0087$). **(Right)** Histogram of mutation rates on random images (red) is shifted to the right compared to a histogram of mutation rates on MNIST images (blue).

Token	Index
Line break	0
’	1
(	2
)	3
,	4
-	5
.	6
0	7
1	8
2	9
3	10
4	11
5	12
6	13
7	14
8	15
9	16
=	17
BatchNormalization	18
Conv1D	19

Token	Index
Conv2D	20
Dense	21
MaxPool2D	22
Reshape	23
X_layer	24
[	25
]	26
activation	27
con	28
concatenate	29
filters	30
g_layer	31
kernel_size	32
loss_weight	33
pool_size	34
relu	35
sigmoid	36
strides	37
units	38
PAD	39

Table A. *RiboAE* source-code token mapping. This mapping was used to translate Python source-code tokens to integers and integers to Python source-code tokens for pre- and post-processing the input and output of the *RiboAE*, respectively.

	Fertility (±SEM)	Mutation rate (±SEM)	Offspring survival rate (±SEM)	# Neurons in next-to-last layer (±SEM)
m_{37}	$0.941\ (\pm 10^{-5})$	$0.32\ (\pm 10^{-3})$	0.93 (±0.00021)	186.8 (±0.009)
m_{83}	$0.941\ (\pm 10^{-5})$	$0.14\ (\pm 4 \times 10^{-4})$	0.98 (±0.00006)	195.3 (±0.003)
m_{37}+m_{83}	$0.942\ (\pm 7 \times 10^{-5})$	$0.77\ (\pm 3 \times 10^{-2})$	0.47 (±0.00311)	160.7 (±0.3)

Table B. Fertility, mutation rate, offspring survival rate, and number of neurons in genotypes carrying *m37* and *m83*. Values shown are averaged over all genotypes that carried only *m37* or *m83* or both (*m37+m83*). In each evaluation, the mutation rate and offspring survival rate were measured with 100 genotype self-replications, and fertility was determined on the entire classification evaluation set (3,000 examples). Only 0.6% of genotypes carrying *m83* also carried *w37* during our evolutionary experiment. All values (except # neurons) are averaged over 50 evaluations per genotype. SEM: standard error of the mean.

Epistasis type		Condition	# quartets	
Additive		$g_{37-83} = g_{37} + g_{83} - g_{wt} \pm \epsilon$	9	
Positive	Sign	$g_{37} < g_{wt} - \epsilon$ and $g_{83} < g_{37-83} - \epsilon$<br>$g_{83} < g_{wt} - \epsilon$ and $g_{37} < g_{37-83} - \epsilon$	10	21
	Magnitude	$g_{37-83} > g_{37} + g_{83} - g_{wt} + \epsilon$	11	
Negative	Sign	$g_{37} > g_{wt} + \epsilon$ and $g_{83} > g_{37-83} + \epsilon$<br>$g_{83} > g_{wt} + \epsilon$ and $g_{37} > g_{37-83} + \epsilon$	11	19
	Magnitude	$g_{37-83} < g_{37} + g_{83} - g_{wt} - \epsilon$	8	

Table C. Epistatic interaction types between *m37* and *m83*. Fertility measured on 49 quartets of genotypes (wild-type, single mutants, and double mutant). Here, $\varepsilon = 0.001$ is the fertility evaluation margin-of-error that is used to distinguish between measurement error and significant deviation. In additive epistasis, the effect of the double mutation is the sum of the effects of the single mutations. In sign epistasis, one mutation has the opposite effect when in the presence of another mutation. In magnitude epistasis, the effect of the double mutation is greater than the sum of the effects of the single mutations.

	X	X_Dense	X_MaxPool2D	X_Conv2D	X_BatchNorm	g	g_Dense	g_Conv1D	g_BatchNorm	concat	M_Dense	M_BatchNorm	outputs
X	0	0.1	0.1	0.6	0	0	0	0	0	0.2	0	0	0
X_Dense	0	0.1	0	0.2	0.2	0	0	0	0	0.5	0	0	0
X_MaxPool2D	0	0.3	0	0.4	0	0	0	0	0	0.3	0	0	0
X_Conv2D	0	0.1	0.4	0.2	0.2	0	0	0	0	0.1	0	0	0
X_BatchNorm	0	0.2	0.1	0	0	0	0	0	0	0.7	0	0	0
g	0	0	0	0	0	0	0.4	0.4	0	0.2	0	0	0
g_Dense	0	0	0	0	0	0	0.1	0.2	0.2	0.5	0	0	0
g_Conv1D	0	0	0	0	0	0	0.3	0.2	0.2	0.3	0	0	0
g_BatchNorm	0	0	0	0	0	0	0.2	0.15	0	0.65	0	0	0
concat	0	0	0	0	0	0	0	0	0	0	1	0	0
M_Dense	0	0	0	0	0	0	0	0	0	0	0.2	0.2	0.6
M_BatchNorm	0	0	0	0	0	0	0	0	0	0	0.1	0	0.9

Table D. Transition probabilities of the SeRANN source code generator. This matrix was used to generate the synthetic SeRANN data set. The value in row *i* and column *j* is the probability that layer *j* comes after layer *i*.

	Mutant allele			Dominant genotype			
**Site**	Appearance generation	Fixation generation	Fixation time	Absolute fitness	Parent absolute fitness	Relative fitness	Phenotype difference from parent
**65**	1	26	25	0.623	0.641	1.52	*synonymous*
**31**	1	42	41	0.837	0.782	1.2	*synonymous*
**71**[*]	1	52	51	0.641	0.492	1.17	*synonymous*
**85**[*]	1	55	54				
**34**	1	182	181				
**77**	1	57	56	0.867	0.891	1.14	strides in classification layer 2->1
**6**	1	76	75	0.883	0.623	1.2	-0.05 in loss_weight; +40 neurons in next-to-last layer
**35**	1	173	172	0.867	0.131	1.16	-0.2 loss_weight; +30 neurons in next-to-last layer
**91**	1	174	173	0.764	0.883	1.43	-30 neurons in next-to-last layer; +0.002 in loss_weight
**38**	1	179	178	0.897	0.867	0.99	# conv filters in classification layer 32->16
**11**	1	195	194	0.937	0.897	1.02	+39 neurons in next-to-last layer
**37**	1	195	194	0.782	0.763	1.22	-0.0022 loss_weight
**66**	1	270	269	0.93	0.837	1.27	kernel size in classification layer 5->7; +30 neurons in next-to-last layer
**84**	1	1351	1350	0.943	0.937	1.05	strides in classification layer 2->1; # conv filters in classification layer 8->32; # conv filters in replication layer 16->8
**47**[†]	191	1420	1229	0.936	0.06	1.05	+0.002 in loss_weight; convolution layer switched back from classification to replication; see fig D.
**22**[†]	1	1420	1419				
**83**[†]	804	1439	635				
**3**[‡]	655	1792	1137	0.929	0.935	1.001	+10 more neurons in next-to-last layer
**62**[‡]	1486	2426	940				
**44**	1791	2650	859	0.947	0.938	1.02	*synonymous*
**64**	2693	3357	664	0.935	0.947	1.02	*synonymous*
**53**	3126	3788	662	0.94	0.923	1.01	-0.002 in loss_weight
**45**	4060	4711	651	0.938	0.934	1.01	-0.002 in loss_weight
**59**	4333	5307	974	0.938	0.939	1.01	*synonymous*

Table E. Mutant allele fixations. Details for mutant alleles that reached fixation in the population, ordered by fixation generation, and their dominant genotypes—the genotype that carries the mutant allele and had the largest number of descendants. *Appearance generation* is the generation at which the mutant allele appeared before going to fixation (i.e., it did not disappear before going to fixation). *Fixation generation* is the generation in which the mutant allele first reached 90% frequency. *Fixation time* is the difference between appearance generation and fixation generation. *Fitness* estimates are for the *dominant genotype*—the genotype that carries the mutant allele and had the largest number of descendants. *Relative fitness* is relative to the population mean fitness at appearance generation. *Phenotype difference from parent* is the source code difference between the dominant genotype and its parent; a *synonymous* mutation did not change the source code compared to the parent.
* † ‡ These mutant alleles share the same dominant genotype.

Genotype	Loss Weight	Mutation rate	Fertility
g62	0.1086	0.04987	0.94153
g62-44	0.1086	0.04188	**0.94156**
g62-44-64	0.1086	0.04111	0.94151
g62-44-64-53	0.1066	0.04023	0.94097
g62-44-64-53-45	0.1046	0.03502	0.94035
g62-44-64-53-45-59	0.1046	**0.03374**	0.94026
g62-44-64-99	0.1086	*0.17554*	0.93999
g62-44-64-99-53	0.1066	*0.16581*	0.93945

Table F. *g62* lineage. Details for seven mutant genotypes in the *g62* lineage. See Fig 4 for genotype frequencies. The mutation rate of *g62-44* is significantly lower than that of *g62* (effect size 16.02%; two-sided t-test, $t \approx -6 \times 10^{16}$, $P \approx 0$).
